# Discovery of New Synthetic Routes of Amino Acids in Prebiotic Chemistry

**DOI:** 10.1021/jacsau.4c00685

**Published:** 2024-11-18

**Authors:** Xiao-Tian Li, Sixuan Mi, Yuzhi Xu, Bo-Wen Li, Tong Zhu, John Z. H. Zhang

**Affiliations:** † Faculty of Synthetic Biology, Shenzhen University of Advanced Technology, Shenzhen 518055, China; ‡ Shanghai Engineering Research Center of Molecular Therapeutics and New Drug Development, School of Chemistry and Molecular Engineering, East China Normal University, Shanghai 200062, China; § NYU-ECNU Center for Computational Chemistry at NYU Shanghai, Shanghai 200062, China; ∥ Department of Chemistry, New York University, New York, New York 10003, United States; ⊥ Collaborative Innovation Center of Extreme Optics, Shanxi University, Taiyuan 030006, Shanxi, China; # Shanghai Innovation Institute, Shanghai 200003, China

**Keywords:** RTIP, prebiotic synthesis of amino acid, origin of life, reaction pathway sampling, chemical space exploration

## Abstract

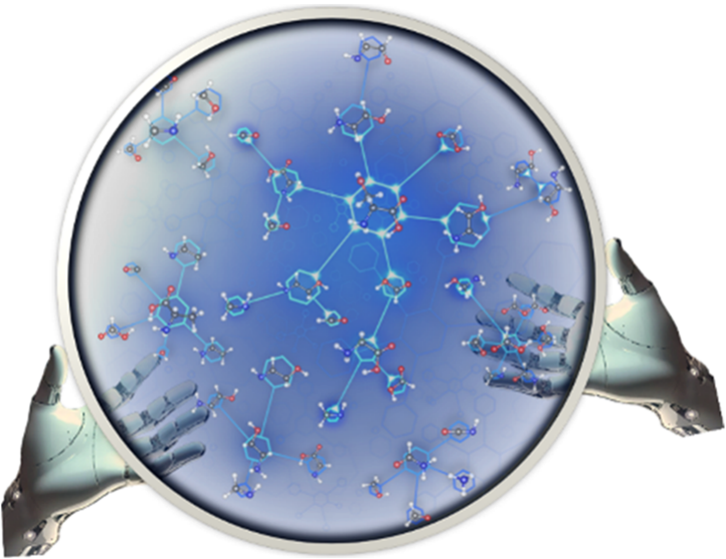

The origin of life on Earth remains one of the most perplexing challenges in biochemistry. While numerous bottom-up experiments under prebiotic conditions have provided valuable insights into the spontaneous chemical genesis of life, there remains a significant gap in the theoretical understanding of the complex reaction processes involved. In this study, we propose a novel approach using a roto-translationally invariant potential (RTIP) formulated with pristine Cartesian coordinates to facilitate the simulation of chemical reactions. By employing RTIP pathway sampling to explore the reactivity of primitive molecules, we identified several low-energy reaction mechanisms, such as two-hydrogen-transfer hydrogenation and HCOOH-catalyzed hydration and amination. This led to the construction of a comprehensive reaction network, illustrating the synthesis pathways for glycine, serine, and alanine. Further thermodynamic analysis highlights the pivotal role of formaldimine as a key precursor in amino acid synthesis, owing to its more favorable reactivity in coupling reactions compared to the traditionally recognized hydrogen cyanide. Our study demonstrates that the RTIP methodology, coupled with a divide-and-conquer strategy, provides new insights into the simulation of complex reaction processes, offering promising applications for advancing organic design and synthesis.

## Introduction

Within the vast and intricate chronicles of the earth, the unique advent of life appears to be an enigmatic puzzle, despite the numerous hypotheses put forth over decades of ongoing efforts.^1−5^ Among them, the pioneering Urey-Miller experiment,^1^ which surprisingly detected the spontaneous formation of some vital biotic molecules, notably amino acids, from a mixture of reducing gases (i.e., H_2_, CH_4_, NH_3_, and H_2_O) in a spark discharge, inaugurated the modern era in prebiotic chemistry research. Following Urey-Miller experiment, more other biologically significant molecules, such as adenine^6^ and cyanoacetylene,^7^ were successively synthesized in a reducing atmosphere, laying the foundation of the prebiotic soup hypothesis.^8^ While these model experiments have provided a rationale for life’s spontaneous chemical emergence, the complex details remain to be elucidated that how these primordial molecules systematically assemble to construct the biotic building blocks in a stepwise manner, which however, is challenging for experiments to observe and identify.^1,9−13^ Furthermore, although traditional quantum chemical calculations based on preset reaction coordinates have undeniably yielded significant insights into the structures and energetics for some potential synthetic pathways,^14−22^ the breadth of our understanding remains limited by the presumed reaction mechanisms. Advanced techniques are requisite for an exhaustive investigation on the complex reaction network.

As one of contemporary challenges in computational chemistry, advancing the theoretical predictive capacity to unlock novel compounds and cost-efficient synthetic pathways has attracted substantial research interest.^23−25^ Throughout recent years, the field witnessed the proposal of diverse intelligent methodologies on pathway sampling and reaction exploration, encompassing the approaches based on molecular simulation,^26−28^ induced force,^29−32^ connectivity graph,^33−35^ and reaction template,^36^ yielding new insights into prebiotic chemistry. For instance, by utilizing a forward-synthesis algorithm grounded in chemist-coded rules, decision tree, and network analyses,^37^ Wołos et al. recently presented a multistep network delineating the evolution process of six primordial molecules (H_2_O, N_2_, HCN, NH_3_, CH_4_, and H_2_S) into a diverse spectrum of biotic and abiotic products.^38^ However, this network has not incorporated quantum chemical calculations to examine the elementary conversions and their associated energetics, which are crucial for a thorough kinetic and thermodynamic analysis. A paradigmatic approach providing such in-depth insights is the *ab initio* nanoreactor (AINR) proposed by Wang et al.,^26^ where a high temperature (e.g., 2000 K) coupled with a periodic piston compression are employed to enhance the reaction rate in *ab initio* molecular dynamics. Based on AINR, Wang et al. traced the synthetic routes of glycine from CO, H_2_, H_2_O, and NH_3_,^26^ while Das et al. revealed that hydrogen cyanide (HCN) and H_2_O can also yield a suite of biomolecular precursors, including glycine, glycolaldehyde, cyanamide, and oxazole derivative.^39^ Later, Zhao et al. revisited the reaction network originating from HCN and H_2_O by using the yet another reaction program (YARP) method,^40^ an exhaustive reaction enumeration algorithm relying on the bond-electron matrix.^35,41,42^ Concentrating on the b2f2 reactions (involving two bonds breaking and two bonds forming) and the H_2_O-catalyzed b3f3 reactions, Zhao et al. reported a more comprehensive reaction network and elucidated the important catalytic role of H_2_O.^40^

Nevertheless, despite the significant advancements achieved by these studies, a question persists concerning the potential oversight of some key reaction mechanisms by current reaction exploration algorithms. Based on molecular dynamics, AINR is closer to reality involving the effect of stereochemistry, stoichiometric ratio, as well as the long-range interaction from surrounding molecules. As it naturally traces the most likely reactions facilitated by high temperature and periodic piston compression, some rare reactions may be overlooked, e.g., those limited by a low concentration of reactants. On the contrary, YARP can enumerate all potential reactions by traversing the bond-electron matrix. However, this approach leads to an exponential growth in the reaction network, particularly when dealing with many-body reactions, thereby limiting its ability to deeply explore the formation of overly complex products. For rationalizing the chemical origin of life, more comprehensive methods turn out to be highly desired to supplement and refine the explosive network, encompassing the biotic building blocks, self-replicating entities, and ultimately the primitive organism,^13,43−46^ which requires a generational research effort.

In this study, we present an automated and highly efficient pathway sampling technique for comprehensive exploration of the reactivity of organic molecules originating from primordial CO, H_2_, H_2_O, and NH_3_. Similar to YARP, this method focuses on the reactivity of selected molecules and overlooks some other details, such as stoichiometric ratios and long-range interactions. The difference is that our method constructs a roto-translationally invariant potential to push molecules closer for potential reactions, which is inferior to YARP’s enumeration method in broad exploration but excels in deep exploration, and it is also more suitable for handling multimolecule reactions. This allows for the construction of low-energy reaction networks, which involve several novel reaction mechanisms, and showcase the diverse synthetic pathways toward glycine, serine, and alanine. Based on thermodynamic correction, the free energy profiles for the competing synthetic pathways are subsequently ascertained. The results indicate that, formaldimine serves as a pivotal precursor in amino acid synthesis due to its more favorable reactivity in coupling reactions, compared to the well-known hydrogen cyanide. These findings provide new insights into prebiotic chemistry and enhance our comprehension of the origin of life.

## Results

### Architecture of Theoretical Methodologies and Models

To elucidate the complex reaction mechanisms in prebiotic chemistry, our studies commenced with the reactivity of foundational molecules, including primordial CO, H_2_, H_2_O, NH_3_, along with their most elementary derivatives (i.e., the C1 species). This was achieved by our newly developed pathway sampling approach, as propelled by an incrementally escalating virtual potential. Uniquely, our proposed virtual potential was formulated using a metric derived from pristine Cartesian coordinates, eschewing the traditional dependency on specific internal coordinates, such as interatomic distances and angles. As a result, the search direction could be flexibly regulated to allow for automated and efficient pathway sampling. Significantly, we substantiated that any function formulated from this metric preserves invariance under roto-translational symmetry, thereby adhering to the fundamental conservation laws in physics. For a detailed introduction to the approach, specifically the roto-translationally invariant potential (RTIP), please refer to the Methods section.

**Figure 1:**
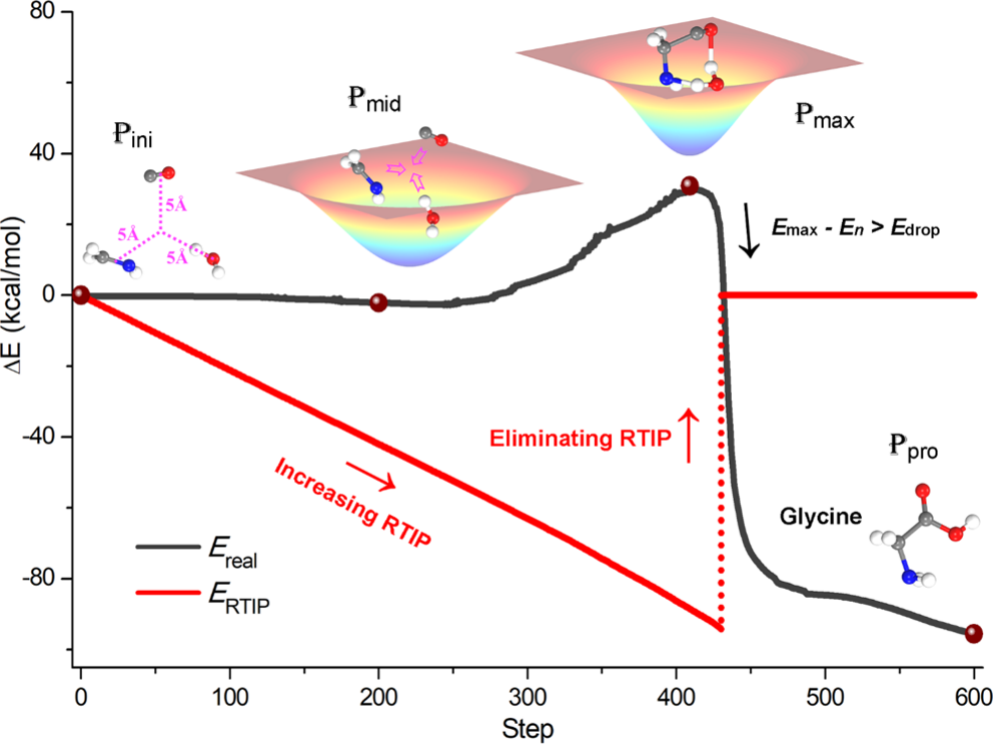
Schematic diagram of the RTIP pathway sampling. Initially, a configuration denoted as ***P***_ini_, is constructed by the stochastic distribution of the reactive molecules, each positioned at a distance of 5 Å from the centroid. Next, a Gaussian-type attractive RTIP is superimposed onto the real potential energy surface (PES), with the potential energies associated with RTIP and real PES denoted by the red line and the black line, respectively. During the exploration, the depth of RTIP increases linearly with the searching step, effectively pushing the molecules closer toward for potential reactions, while the real PES applied on the molecules determines their ultimate reactivity. Once the configuration (e.g., ***P***_max_) surpasses the reaction transition state (TS), it is expected to detect a significant decrease in the real potential energy due to the formation of new chemical bonds (*E*_max_ – *E*_*n*_ > *E*_drop_, where *E*_drop_ = 12.5 kcal mol^–1^ is the predefined threshold in this study). Thereafter, the RTIP would be eliminated and a local optimization is conducted based on the real PES, guiding the configuration toward the desired product ***P***_pro_.

The outline of the RTIP method, as proposed in this work, is illustrated in Figure 1, where a Gaussian-type attractive RTIP with an increasing depth is introduced to gradually push the reactive molecules closer together for potential reactions. Upon the detection of a significant decrease in the real potential energy, which indicates the establishment of new chemical bonds, the RTIP would be eliminated immediately, and instead, a local optimization is carried out to yield the final products. To achieve a balance between computational efficiency and accuracy, the RTIP pathway sampling was performed based on the semiempirical extended tight binding model (GFN0-*x*TB),^47,48^ while the key structures, energetics, and thermodynamic properties were further validated by higher-precision density functional theory (DFT) calculations (i.e., ωB97M-V/TZVP).^49−51^ For more details, please see the Methods section.

Admittedly, our theoretical methods and models exhibit certain limitations, as elaborated below. In Urey-Miller experiment, a gaseous mixture abundant in steam was injected into a sparking chamber to simulate the spark discharge synthesis of organic molecules by lightning.^1,9,10^ This suggests that the reactions initially proceeded in the gas phase, whereas the intermediate species may undergo solubilization at certain junctures, enabling the downstream reactions. For simplicity, all reactions in this study are calculated exclusively in the gas phase. Therefore, it is important to note that the inclusion of solvation effect may induce variations in the structures and energetics for the reactions in solution. Besides, the spark discharge delivered a substantial amount of energy into the system, potentially facilitating the high-energy reactions and resulting in the formation of a wide array of unstable species, like radicals. Upon our analysis on reaction energetics, the vast majority of unstable species do not predominate within the synthetic pathways as compared to the most stable allotropes; instead, they appear to significantly amplify the complexity of the reaction network. The illustrative cases are dihydroxycarbene (COHOH*) and formamidic acid (NHCHOH), which exhibit a degree of chemical equivalence to the more readily synthesized analogs, formic acid (HCOOH) and formamide (HCONH_2_). In this study, a selection of typical molecules is given precedence to promote the in-depth exploration, while the functionally analogous but unstable species, including a vast array of radicals, have been deliberately excluded. As a consequence, the real reaction network could be substantially broader than what is delineated in the current study. Lastly, it is critical to highlight that, the reactions documented herein primarily exhibit elevated activation free energy barriers, which however, aligns coherently with the crucial role of spark discharge in the Urey-Miller experiment, as well as the extensive time scale associated with the emergence of life.

### Genesis of Fundamental Organic Molecules

**Figure 2:**
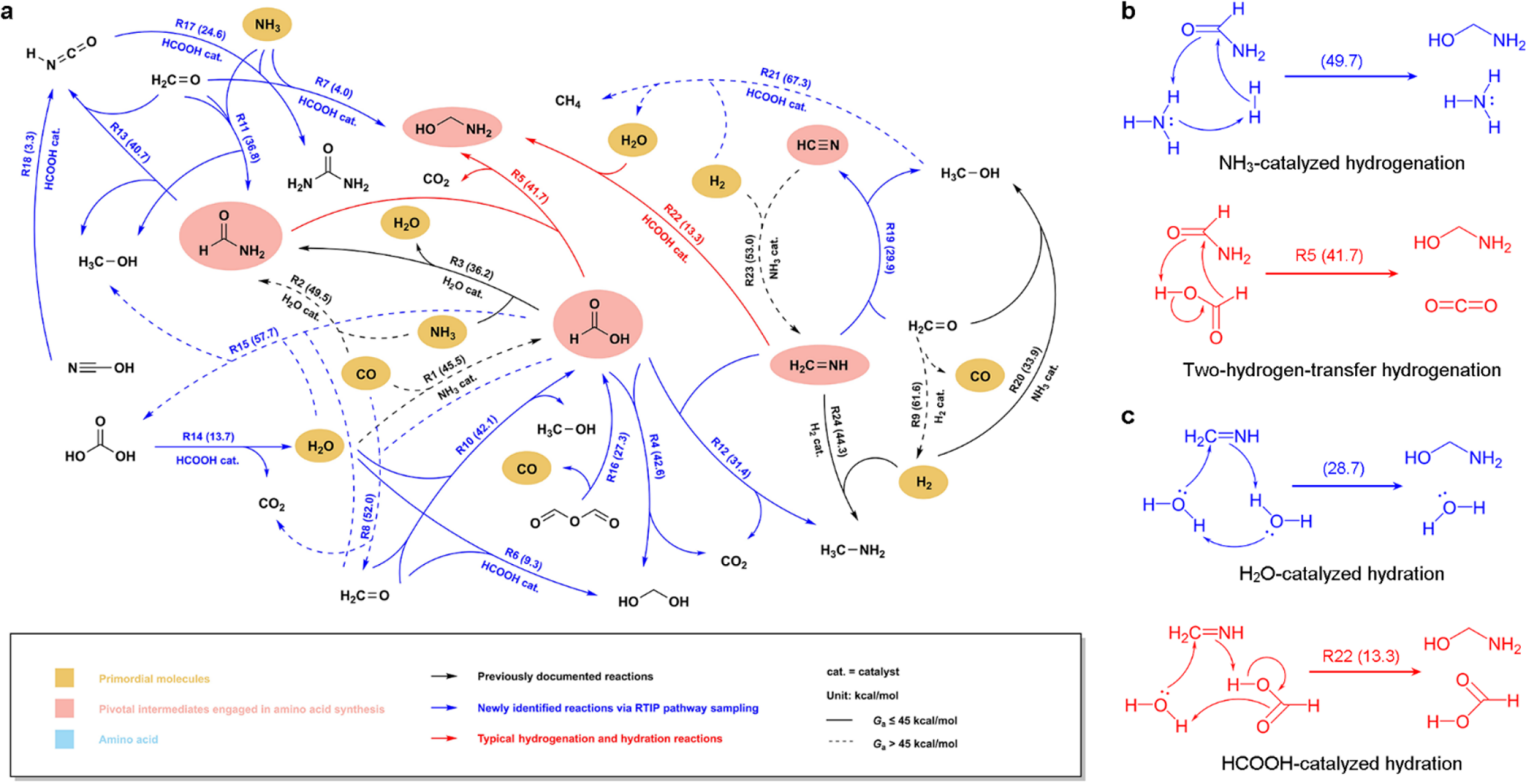
Unbiased reaction network and typical reaction mechanisms in prebiotic chemistry. (a) Low-energy reaction network explored by unbiased RTIP pathway sampling and validated by DFT calculations with ωB97M-V functionals and thermodynamic correction (at 298 K and 1 atm). The network interconnects primordial molecules CO, H_2_, H_2_O, and NH_3_ (underpainted in yellow), as well as the pivotal derivatives in amino acid synthesis (underpainted in tomato), with each arrow representing an elementary reaction directed toward the exothermic pathway (not directed toward the actual reaction process). For clarity, each elementary reaction is annotated with an index, accompanied by its activation free energy barrier in parentheses (*G*_a_, in kcal mol^–1^), while the less viable reactions with *G*_a_ > 45 kcal mol^–1^ are distinctly delineated by dotted lines. (b, c) Detailed mechanism for typical hydrogenation and hydration reactions in prebiotic chemistry.

By applying the Gaussian-type attractive RTIP on primordial molecules and their elementary derivatives, we conducted a comprehensive investigation into the reaction space in prebiotic chemistry. This unveiled a spectrum of organic molecules that emerge at the early stages, as summarized in the unbiased low-energy reaction network in Figure 2a (please refer to Supporting Table S1 for the detailed information of the elementary reactions). A multitude of elementary reactions have been newly identified, exhibiting lower Gibbs free energy barriers compared to previously reported ones,^26,39,40^ as distinguished by blue and black arrows in the reaction network, respectively. Typically, the prebiotic chemistry originates from two similar reactions, namely NH_3_-catalyzed synthesis of formic acid (HCOOH) from CO and H_2_O (R1, *G*_a_ = 45.5 kcal mol^–1^), and H_2_O-catalyzed synthesis of formamide (HCONH_2_) from CO and NH_3_ (R2, *G*_a_ = 49.5 kcal mol^–1^). In addition to direct synthesis, formamide can also be generated from formic acid by substituting an amino group for a hydroxyl group as catalyzed by a H_2_O molecule, which exhibits a significantly lower free energy barrier of 36.2 kcal mol^–1^ (R3). The early synthesized formic acid and formamide can undergo two-hydrogen-transfer hydrogenation as facilitated by a formic acid molecule, to yield methanediol (CH_2_(OH)_2_) and aminomethanol (NH_2_CH_2_OH), respectively (R4–5, *G*_a_ = 42.6 and 41.7 kcal mol^–1^), which will be elaborated upon in the subsequent discussion. As catalyzed by a formic acid molecule, methanediol and aminomethanol can further undergo dehydration and deamination to achieve formaldehyde (CH_2_O, R6–7, *G*_a_ = 9.3 and 4.0 kcal mol^–1^), an important building block in prebiotic chemistry. In a similar manner, aminomethanol can also undergo HCOOH-catalyzed dehydration, leading to the formation of formaldimine (CH_2_NH, R22), which can subsequently dehydrogenate to produce hydrogen cyanide (HCN) (R19, *G*_a_ = 29.9 kcal mol^–1^). Formaldimine and hydrogen cyanide turn out to be the most important precursors for the synthesis of amino acids, as will be elucidated in the next section.

According to our DFT theoretical calculations, hydrogenation and dehydrogenation reactions, which are crucial for regulating species saturability in prebiotic chemistry, typically exhibit moderate free energy barriers ranging from 30 to 45 kcal mol^–1^. Previously, a commonly acknowledged reaction mechanism is the NH_3_-catalyzed hydrogenation by H_2_,^26,39^ wherein an ammonia molecule participates as the proton shuttle for reducing the barrier. As a representative, the hydrogenation mechanisms of formamide are illustrated in Figure 2b-1, where the NH_3_-catalyzed hydrogenation exhibits a relatively lower free energy barrier of 49.7 kcal mol^–1^ compared to the direct hydrogenation by H_2_ (*G*_a_ = 69.9 kcal mol^–1^, see Supporting Table S1). Nevertheless, our RTIP pathway sampling has discerned a distinctive mechanism governing hydrogenation reactions within prebiotic chemistry, namely two-hydrogen-transfer hydrogenation/dehydrogenation, which was initially presented by Zimmerman et al. for CO_2_ reduction.^52^ As exemplified in Figure 2b-2, the concerted transfer of two hydrogen atoms from formic acid to formamide enables the simultaneous hydrogenation of formamide and dehydrogenation of formic acid, with the lowest free energy barrier of 41.7 kcal mol^–1^. Such two-hydrogen-transfer reactions involving two or three molecules are prevalent in prebiotic chemistry, mediating the conversions between a wide array of species, including formic acid and carbon dioxide (CO_2_, R12, *G*_a_ = 31.4 kcal mol^–1^), formamide and isocyanic acid (HNCO, R13, *G*_a_ = 40.7 kcal mol^–1^), CO and formaldehyde (R8, *G*_a_ = 52.0 kcal mol^–1^), formaldimine and hydrogen cyanide (R19, *G*_a_ = 29.9 kcal mol^–1^), H_2_O, formaldehyde and formic acid (R10, *G*_a_ = 42.1 kcal mol^–1^), NH_3_, formaldehyde and formamide (R11, *G*_a_ = 36.8 kcal mol^–1^), and so on. Among these species, formic acid and formaldimine display a pronounced propensity to donate two hydrogen atoms for the saturation of a double or triple bond, positioning them as predominant hydrogenating agents in contrast to H_2_ as catalyzed by NH_3_ (please refer to Table 1 for the comparison).

**Table 1:** Activation Free Energy Barriers (*G*_a_, in kcal mol^–1^) for Typical Mechanisms in Several Hydrogenation, Hydration, and Amination Conversions

Reaction type	Conversion	Agent, catalyst and free energy barrier^a^		
		+H_2_, NH_3_ cat.	+HCOOH – CO_2_	+CH_2_NH – HCN
Hydrogenation		33.9	31.4	**29.9**
		53.0	42.2	**41.2**
		46.2	**31.4**	31.8
		45.5	**42.6**	51.3
		49.7	**41.7**	50.7
		H_2_O cat.	NH_3_ cat.	HCOOH cat.
Hydration and amination		23.2	23.7	**9.3**
		15.8	21.7	**4.0**
		28.7	34.0	**13.3**
		22.7	20.3	**13.7**
		42.0	40.6	**24.6**

a The free energy barriers were calculated by DFT with ωB97M-V functionals and thermodynamic correction (at 298 K and 1 atm), with the lowest barriers highlighted in bold.

In addition to two-hydrogen-transfer hydrogenation, our RTIP approach has also unveiled the versatile catalytic role of formic acid across a spectrum of reactions. For instance, as depicted in Figure 2c, formic acid demonstrates superior efficiency as a proton shuttle compared to H_2_O within the hydration of formaldimine, as evidenced by the sharp reduction in free energy barrier from 28.7 to 13.3 kcal mol^–1^. This phenomenon showcases universality across typical hydration and amination reactions (R6, 7, 14, 17, 22), as demonstrated by the summary presented in Table 1 for the conversions catalyzed by H_2_O, NH_3_, and HCOOH, respectively. Furthermore, formic acid also exhibits catalytic activity in mediating conversions among several other compounds, including isocyanic acid and cyanic acid (NCOH, R18, *G*_a_ = 3.3 kcal mol^–1^), as well as methanol (CH_3_OH) and methane (CH_4_, R21, *G*_a_ = 67.3 kcal mol^–1^). Within the prevailing literature, there is consensus that H_2_O and NH_3_ serve as crucial proton shuttles in a variety of elementary reactions.^26,39,40^ Here we complement the vital catalytic role of formic acid, thereby enriching our understanding to help unveil novel synthetic pathways toward biologically significant molecules.

### Directed Network toward Amino Acids

**Figure 3:**
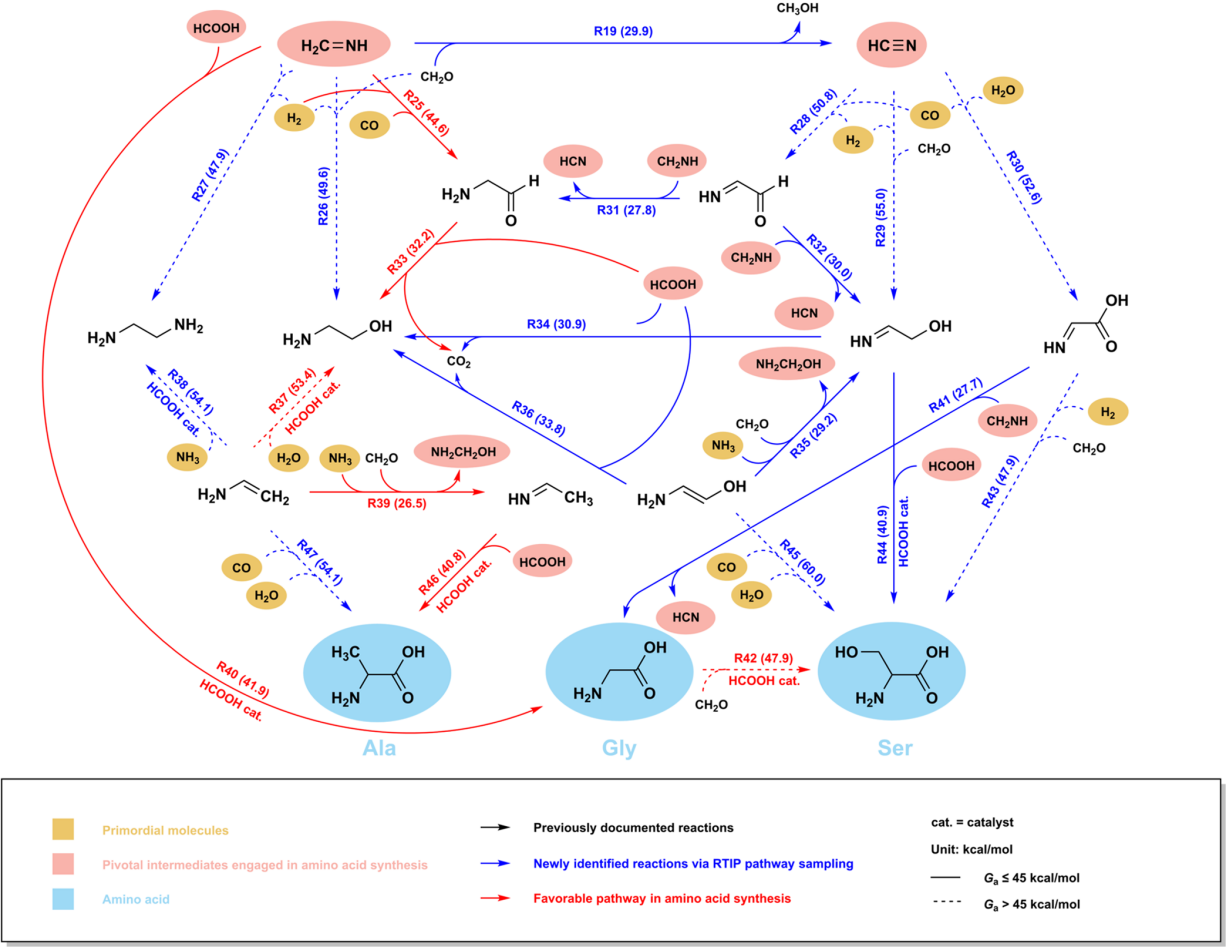
Reaction network directed toward glycine, serine, and alanine. The low-energy reaction network was explored by RTIP pathway sampling directed toward amino acids, and validated by DFT calculations with ωB97M-V functionals and thermodynamic correction (at 298 K and 1 atm). The consistency of signage persists, as depicted in Figure 2a, with specific red arrows highlighting the most favorable pathways in amino acid synthesis (please refer to Figure 4 for comprehensive details).

Based on the robust RTIP approach, we have established a diverse network, even inclusive of several inaccessible products due to either considerable endothermicity (e.g., R14, 16, 18 for the synthesis of carbonic acid, formic anhydride, and cyanic acid) or relatively high barrier (e.g., R21 for the synthesis of methane). Next, in pursuit of in-depth exploration, we strategically directed our pathway sampling toward the precursors of amino acids, yielding a low-energy reaction network summarized in Figure 3. Evidently, formaldimine and hydrogen cyanide emerged as pivotal precursors in amino acid synthesis, attributed to their unsaturated C–N bonds, which serve as reactive sites enabling subsequent coupling reactions. For instance, upon cleaving one of its C=N double bonds, formaldimine can couple with CO and H_2_ to yield 2-aminoacetaldehyde (NH_2_CH_2_CHO, R25), with H_2_ and formaldehyde to yield ethanolamine (NH_2_CH_2_CH_2_OH, R26), with H_2_ and another formaldimine molecule to yield ethylenediamine (NH_2_CH_2_CH_2_NH_2_, R27). Similarly, hydrogen cyanide can undergo coupling reactions with CO and H_2_ to yield aminoketene (NHCHCHO, R28), with H_2_ and formaldehyde to yield iminoethanol (NHCHCH_2_OH, R29), with CO and H_2_O to yield iminoglycine (NHCHCOOH, R30). It is crucial to underscore the indispensable role of H_2_ at this juncture, distinct from its nonessential contribution in hydrogenation reactions relative to formic acid and formaldimine. Of particular significance, the coupling reactions involving formaldimine exhibit significantly lower free energy barriers (*G*_a_ = 44.6–49.6 kcal mol^–1^) compared to those involving hydrogen cyanide (*G*_a_ = 50.8–55.0 kcal mol^–1^), thereby exerting a noteworthy impact on the synthesis of amino acids.

After the coupling reactions, the resultant compounds, specifically aminoketene, iminoethanol, 2-aminoacetaldehyde, ethanolamine, and 2-aminovinyl alcohol (NH_2_CHCHOH), demonstrate a moderate propensity for interconversion via the two-hydrogen-transfer mechanism, as facilitated by formic acid or formaldimine (R31–34, 36, *G*_a_ = 27.8–33.8 kcal mol^–1^). Additionally, ethanolamine and ethylenediamine can undergo dehydration and deamination processes, respectively, to yield vinylamine (NH_2_CHCH_2_), with formic acid serving as the catalytic proton shuttle (R37, 38). Afterward, vinylamine can further isomerize into its allotrope, ethanimine (NHCHCH_3_), accompanied by the amination of formaldehyde (R39, *G*_a_ = 26.5 kcal mol^–1^). This is similar to the isomerization between 2-aminovinyl alcohol and iminoethanol (R35, *G*_a_ = 29.2 kcal mol^–1^).

Conclusively, formaldimine and iminoglycine serve as the principal precursors for the synthesis of glycine. Typically, formaldimine can engage in moderate coupling with formic acid to produce glycine, as catalyzed by an additional formic acid molecule (R40, *G*_a_ = 41.9 kcal mol^–1^). This pathway demonstrates a notable decrease in free energy barrier in contrast to a previously documented four-body reaction, i.e., the coupling of formaldimine, CO, and H_2_O, along with another H_2_O molecule as the catalytic proton shuttle (*G*_a_ = 47.0 kcal mol^–1^, see Table S1).^20,26^ Besides, there exists a third noteworthy pathway, namely the two-hydrogen-transfer hydrogenation of iminoglycine, characterized by a pretty low barrier of 27.7 kcal mol^–1^ (R41). Next, in the synthesis of serine, formaldehyde, iminoethanol, iminoglycine, and 2-aminovinyl alcohol turn out to be the designated precursors. Likewise, coupling reactions predominate here, wherein the unsaturated bonds of the precursors can undergo cleavage to enable the synthesis process. The alternative pathways include: HCOOH-catalyzed synthesis from iminoethanol and formic acid (R44, *G*_a_ = 40.9 kcal mol^–1^); HCOOH-catalyzed synthesis from glycine and formaldehyde (R42, *G*_a_ = 47.9 kcal mol^–1^); direct coupling of iminoglycine, H_2_, and formaldehyde (R43, *G*_a_ = 47.9 kcal mol^–1^); and direct coupling of 2-aminovinyl alcohol, CO, and H_2_O (R45, *G*_a_ = 60.0 kcal mol^–1^). Finally, in the synthesis of alanine, ethanimine and vinylamine are identified as essential precursors. Among the two pathways illustrated in Figure 3, the HCOOH-catalyzed synthesis from ethanimine and formic acid (R46, *G*_a_ = 40.8 kcal mol^–1^) is notably more favorable than the direct coupling of vinylamine, CO, and H_2_O (R47, *G*_a_ = 54.1 kcal mol^–1^) due to its significantly lower activation energy. Depicted through the complex reaction network, the synthetic routes toward amino acids showcase a substantial degree of diversity, emphasizing the imperative for further analysis.

### Thermodynamic Analysis on Synthetic Pathways

**Figure 4:**
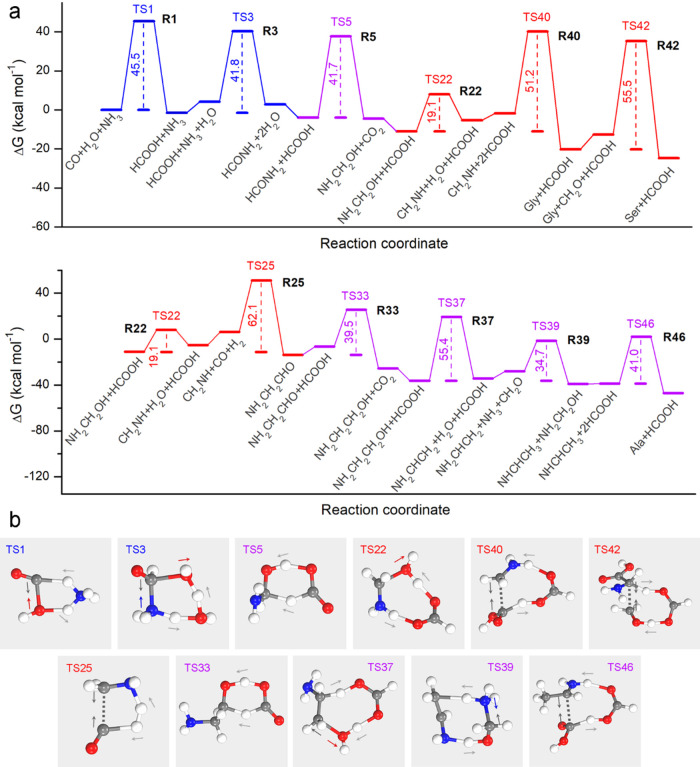
Synthetic pathways for glycine, serine, and alanine. (a) Gibbs free energy profile (at 298 K and 1 atm) for the most favorable synthetic pathways of glycine, serine, and alanine, as determined from the reaction network. For consistency, each elementary step is assigned a unique index that precisely follows the notation employed in the reaction network. The color schemes are as follows: blue denotes previously documented reactions; purple indicates newly identified reactions by RTIP; while red highlights the rate-determining steps for amino acid synthesis. (b) TS structures selected from **a** for illustration of the synthetic pathways. The arrows indicate the vibrational directionality for the atoms involved in bond cleavage and reformation within the imaginary normal mode at TS. Color code: gray ball for C, blue ball for N, red ball for O, white ball for H.

We now proceed to derive potential synthetic pathways for glycine, serine, and alanine from the diverse reaction network, as summarized in Figures 4, S1, and S2. Typically, the reaction commences with the interaction of primordial CO, H_2_O, and NH_3_, leading to the formation of CO–H_2_O–NH_3_ complex. The hydroxyl group of H_2_O then can overcome a Gibbs free energy barrier of 45.5 kcal mol^–1^ to attack CO, along with the concerted transfer of one proton from H_2_O to NH_3_ and another proton from NH_3_ to CO (R1, with corresponding TS1 shown in Figure 4b). This leads to the formation of HCOOH–NH_3_ complex, which is 1.4 kcal mol^–1^ more stable than the CO–H_2_O–NH_3_ complex. Such consecutive exothermic procedures occur in the next two steps, namely H_2_O-catalyzed synthesis of formamide from formic acid and NH_3_, and two-hydrogen-transfer hydrogenation of formamide to aminomethanol, with the reaction barriers being 41.8 and 41.7 kcal mol^–1^, respectively (R3, 5). However, the ensuing HCOOH-catalyzed dehydration of aminomethanol to formaldimine (R22, *G*_a_ = 19.1 kcal mol^–1^) encounters thermodynamic inhibition due to its significant endothermicity of 5.8 kcal mol^–1^. This results in a high free energy barrier of 51.2 kcal mol^–1^ (with respect to NH_2_CH_2_OH–HCOOH complex, the most stable intermediate state along the pathway) for the HCOOH-catalyzed synthesis of glycine, which necessitates formaldimine as the precursor (R40). Subsequently, under the catalysis of formic acid, glycine can overcome a higher barrier of 55.5 kcal mol^–1^ to couple with formaldehyde, leading to the formation of serine. As highlighted in red, the rate-determining steps for glycine synthesis is the dehydration of aminomethanol to formaldimine and the coupling of formaldimine and formic acid (R22, 40), while the rate-determining step for serine synthesis is the coupling of glycine and formaldehyde (R42).

Similarly, the synthetic pathway of alanine also involves the formation of formic acid from CO and H_2_O (R1), the conversion of formic acid to formamide (R3), the hydrogenation of formamide to aminomethanol (R5), and the dehydration of aminomethanol to formaldimine (R22). The rate-determining step occurs in the coupling of formaldimine, CO, and H_2_ to produce 2-aminoacetaldehyde (R25), which exhibits a substantial free energy barrier of 62.1 kcal mol^–1^ relative to NH_2_CH_2_OH–HCOOH complex, the most stable intermediate. Thereafter, 2-aminoacetaldehyde can undergo two-hydrogen-transfer hydrogenation to yield ethanolamine (R33, *G*_a_ = 39.5 kcal mol^–1^), followed by its dehydration to vinylamine as catalyzed by formic acid (R37, *G*_a_ = 55.4 kcal mol^–1^), which can further convert into ethanimine through the transfer of hydrogen atoms (R39, *G*_a_ = 34.7 kcal mol^–1^). Finally, alanine can be synthesized from ethanimine and formic acid as catalyzed by an additional formic acid molecule, which presents a moderate free energy barrier of 41.0 kcal mol^–1^.

Additionally, it is important to acknowledge several alternative synthetic pathways, which are summarized in Supporting Figures S1 and S2. Admittedly, as illustrated by the reaction network in Figure 3, hydrogen cyanide serves as a pivotal precursor in the synthesis of amino acids. However, comprehensive thermodynamic analysis on the entire reaction network (refer to Figures 4, S1, and S2) reveals that, the synthetic pathways via hydrogen cyanide are thermodynamically less favorable compared to those via formaldimine. The illustrative examples include the synthesis of glycine from iminoglycine and the synthesis of serine from aminoketene, both characterized by rate-determining steps in the coupling reactions involving hydrogen cyanide. It shows that the coupling of hydrogen cyanide, CO, and H_2_O (yielding iminoglycine) and the coupling of hydrogen cyanide, CO, and H_2_ (yielding aminoketene) exhibit free energy barriers of 57.7 and 58.6 kcal mol^–1^, respectively, significantly higher than the pathways via formaldimine (51.2 and 55.5 kcal mol^–1^). Besides, although iminoethanol can moderately isomerize into 2-aminovinyl alcohol, the synthesis of serine from 2-aminovinyl alcohol, CO, and H_2_O is also unfavorable, which has a relatively high free energy barrier of 62.8 kcal mol^–1^. Finally, for the synthesis of alanine, vinylamine, as a vital precursor, could also be produced through the deamination of ethylenediamine and the dehydration of ethanolamine. Unfortunately, these pathways are thermodynamically inhibited by the high reaction barriers for the synthesis of ethylenediamine and ethanolamine from formaldimine (64.7 and 67.8 kcal mol^–1^).

We notice that Wang et al., by using AINR, have reported a synthetic pathway of glycine from CO, H_2_, H_2_O, and NH_3_,^26^ which is reproduced by our calculations as shown in Supporting Figure S1. This pathway undergoes the synthesis of formic acid from CO and H_2_O (R1), the hydrogenation of formic acid to methanediol (R4′), the dehydration of methanediol to formaldehyde (R6′), the amination of formaldehyde to aminomethanol (R7′), the dehydration of aminomethanol to formaldimine (R22′), and the final coupling of formaldimine, CO, and H_2_O to achieve glycine (R40′). Throughout this pathway, NH_3_ and H_2_O serve as proton shuttles to facilitate the reactions, exhibiting a favorable free energy barrier of 63.2 kcal mol^–1^. Considering that NH_3_ and H_2_O would be abundant on the primordial earth, this pathway is also feasible to occur.

## Conclusions

In this study, we trace the spontaneous formation of amino acids from a limited array of primordial molecules (CO, H_2_, H_2_O, and NH_3_) using advanced computational simulations, aligned with the landmark Urey-Miller experiment. Utilizing the RTIP pathway sampling method, our study reveals that the two-hydrogen-transfer mechanism is predominant in prebiotic hydrogenation reactions. Additionally, while H_2_O and NH_3_ are widely recognized as important proton shuttles for facilitating various conversions,^26,39,40^ our study demonstrates the superior catalytic capability of formic acid. Based on these novel reaction mechanisms, we have established a comprehensive reaction network that delineates diverse synthetic pathways toward glycine, serine, and alanine, with the lowest rate-determining barriers calculated to be 51.2, 55.5, and 62.1 kcal mol^–1^, respectively. It is noteworthy that these three amino acids closely match the primary products identified in Urey-Miller experiment,^1,11,12^ while the substantial thermodynamic barriers (>50 kcal mol^–1^) elucidate the indispensable role of a high energy flux (e.g., spark discharge or ultraviolet radiation) in facilitating the reactions.^1,13,53,54^

In prebiotic chemistry, there is consensus regarding the pivotal role of hydrogen cyanide as fundamental feedstock for the spontaneous synthesis of a spectrum of biotic molecules.^39,40,55,56^ For instance, the well-established Strecker pathway delineates the synthesis of aminonitriles from hydrogen cyanide and aldehydes, followed by their hydrolysis to yield amino acids.^9,12,13,39^ However, this mechanism fails to adequately explain the simultaneous formation of α- and β-amino acids observed in Urey-Miller experiment.^9,11,12^ Here, based on comprehensive RTIP exploration, our reaction network identifies formaldimine as another pivotal precursor in the synthesis of amino acids, shedding new light on the underlying reaction mechanisms. The validity of this perspective awaits experimental verification.

For a long time, conducting computer-simulated experiments for organic design and synthesis has been a dream. This study presents a divide-and-conquer strategy: the complex reaction process is systematically decomposed into discrete conversions between species, which can be thoroughly explored using the RTIP method. Although certain achievements have been made, there is still room for improvement in this approach. In our future work, we will attempt to introduce machine learning techniques to enable the automatic selection of reactants and the automated analysis of reaction pathways. Additionally, we will explore combining the RTIP method with molecular dynamics simulations to achieve a better description of solvents and long-range interactions. The corresponding results will be presented in our forthcoming studies.

## Methods

### Roto-Translationally Invariant Potential

In chemical space exploration or molecular dynamics simulation, a faithful representation of the atomic interactions is crucial for preserving the system’s symmetry, as governed by the physical conservation laws. While the atomic potentials, ranging from quantum chemistry methods, semiempirical models, and classical force fields, naturally comply with the symmetry constraints as a result of their rigorous theoretical foundations, the virtual potentials potentially face challenges due to their directional specificity for enhanced sampling. Traditionally, certain roto-translational invariants like interatomic distances and angles are chosen to construct the representation space for the virtual potential.^30,57,58^ Such representation spaces, however, appear tedious, demanding meticulous attention to the bond dissociation and reassociation, which limits the predictive capability of the virtual potential.^59^ Here, we present a general potential that preserves rotational and translational symmetry as formulated with Cartesian generalized coordinates, namely RTIP, for enhanced sampling.

In three-dimensional Euclidean space, the geometric configuration of a molecular system consisting of *n* atoms can be represented by tuples of Cartesian coordinates for each atom, denoted as ***R*** = (***r***_1_,···,***r***_*n*_), where ***r***_*i*_ = (*x*_*i*_,*y*_*i*_,*z*_*i*_)*^T^*. For refined representation of spatial transformation, atomic homogeneous coordinates, expressed as ***p***_*i*_ = (*x*_*i*_,*y*_*i*_,*z*_*i*_,1)*^T^*, could be introduced as a substitute for Cartesian coordinates. In this formalism, the roto-translation operation applied to a molecular system ***P*** = (***p***_1_,···,***p**_n_*) is faithfully represented by a 4 × 4 matrix
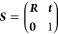
1where ***R*** is the 3 × 3 rotation matrix and  is the translation vector. A metric between two molecular systems ***P***_1_ and ***P***_2_ can be defined as
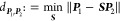
2where ∥ ∥ symbolizes the Frobenius norm (For proofs of non-negativity, symmetry, and triangle inequality of this metric, please refer to the Supporting information). It is worth noting that, this metric manifests roto-translational invariance with respect to both molecular systems, as a consequence of their proper spatial alignment before evaluation of the Frobenius norm (i.e., finding the optimal ***S*** that acts on ***P***_2_ for the minimum norm). More importantly, functions derived from this metric likewise demonstrate roto-translational invariance (see the proof in Supporting information), providing a generalized formulation for the symmetry-conserved virtual potential.

**Figure 5:**
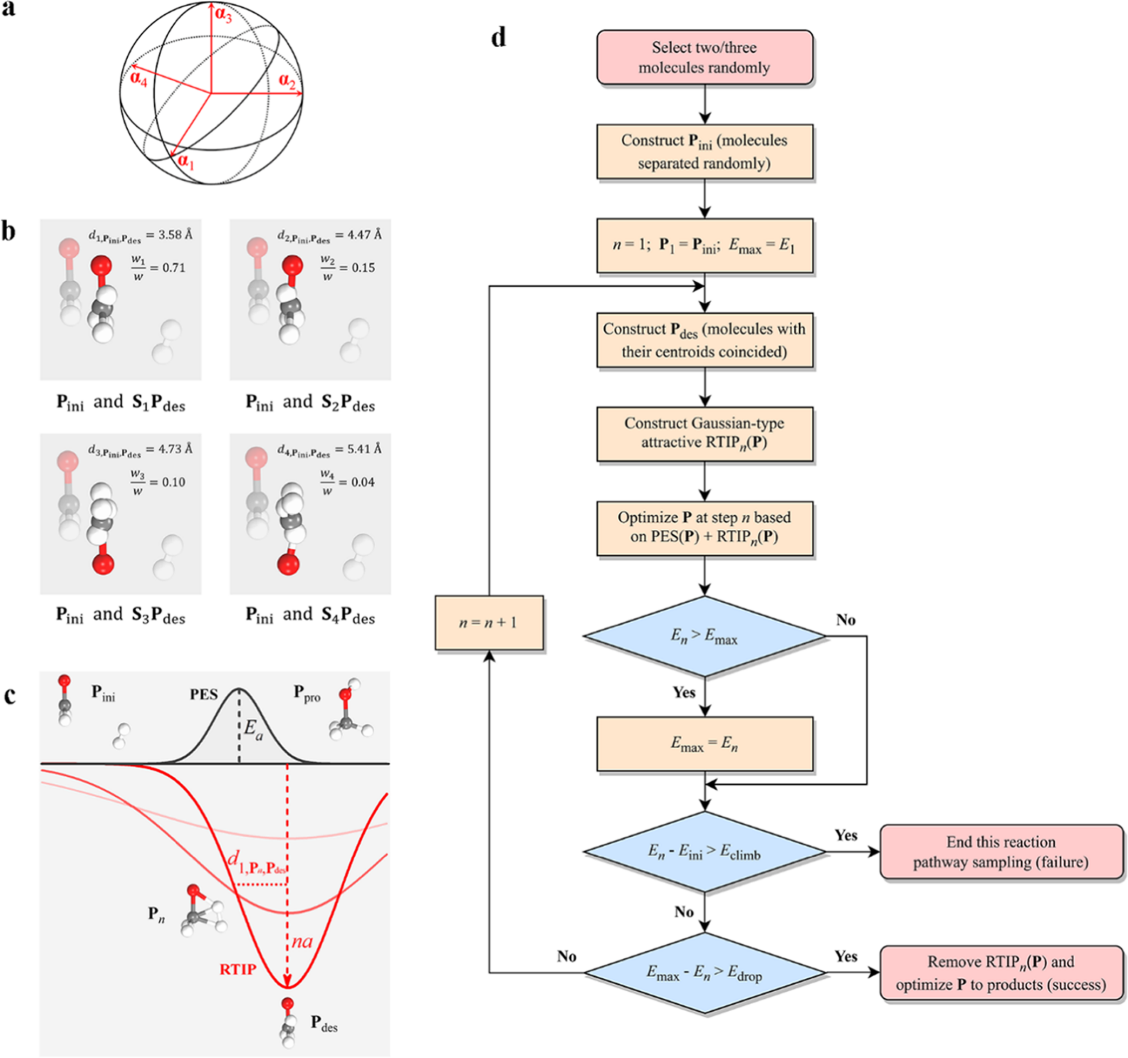
Schematic diagram of RTIP methodology. (a) Least-square fitting of two configurations in quaternion representation, with four orthogonal eigenvectors resolved by spectral factorization. (b) Four corresponding alignment modes between the initial configuration ***P***_ini_ (i.e., the separated formaldehyde and H_2_ in a semitransparent form) and the destination configuration ***P***_des_ (i.e., the coincided formaldehyde and H_2_ in an opaque form) in the direct hydrogenation of formaldehyde. (c) Schematic diagram for the real PES and the attractive RTIP Gaussian function of d_(_1,***P***,***P***_des_), whose depth increases linearly over the searching steps. (d) Flowchart of the RTIP pathway sampling in prebiotic chemistry.

In practical implementations, the optimal translation vector ***t*** can be easily determined by aligning the centroids of ***P***_1_ and ***P***_2_, whereas the optimal rotation matrix ***R*** can be ascertained using the quaternion method.^60^ In quaternion formalism, the least-squares fitting of ***P***_1_ and ***P***_2_ is represented by a 4 × 4 real symmetric matrix, whose spectral factorization yields four eigenvalues, λ_1_ ≤ λ_2_ ≤ λ_3_ ≤ λ_4_, along with their corresponding eigenvectors: **α**_1_, **α**_2_, **α**_3_, **α**_4_ (see Figure 5a). The optimal rotation matrix ***R*** then can be derived from the eigenvector **α**_1_, which is associated with the minimum eigenvalue λ_1_.

In fact, the four eigenvectors delineate four distinct rotation operations, each correlating to a unique alignment mode upon combination with the optimal translation vector ***t***. Significantly, across all the four alignment modes, the difference vector (***P***_1_–***SP***_2_) exhibits inherent orthogonality to the generators of SO(3) group operating on both ***P***_1_ and ***P***_2_. This suggests that the four associated Frobenius norms, denoted as *d*_*i*_,_***P***_1_,***P***_2__ where *i* = 1–4, are intrinsically roto-translationally invariant, rendering them appropriate for the construction of virtual potential. To tackle the nondifferentiability issue arising from degenerate eigenvalues, the RTIP is systematically formulated in a combination manner as follows
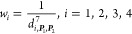
3
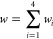
4
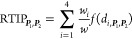
5Herein, the general schema of RTIP bridging ***P***_1_ and ***P***_2_ is defined as the weighted average across a function of *d*_*i*_,_***P***_1_,***P***_2__. When λ_1_ ≪ λ_2_ ≪ λ_3_ ≪ λ_4_, RTIP_***P***_1_,***P***_2__ is primarily governed by *d*_1_,_***P***_1_,***P***_2__, corresponding to the optimal alignment mode. While in degenerate cases, e.g., λ_1_ = λ_2_ ≤ λ_3_ ≤ λ_4_, RTIP_***P***_1_,***P***_2__ is equally impacted by both *d*_1_,_***P***_1_,***P***_2__ and *d*_2_,_***P***_1_,***P***_2__, thereby preventing a discontinuous transition from λ_1_ to λ_2_.

### Gaussian-Type Attractive RTIP

Within the combination framework of RTIP, we now introduce its application for probing the reactivity of primordial molecules in prebiotic chemistry. As depicted in the flowchart in Figure 5d, the pathway sampling is commenced with the random selection of either two or three molecules from the candidate list, allowing for repetition if necessary. Subsequently, the selected molecules are randomly placed in a distance from the centroid (a 5 Å separation is adopted in this study, since the size of the molecules involved is smaller than 5 Å), establishing the initial configuration ***P***_ini_ (as exemplified by the separated formaldehyde and H_2_ in Figure 5c). To investigate the reactivity of the molecules, a virtual structure is deliberately fabricated as the destination configuration ***P***_des_, with the centroids of the molecules coincided (as illustrated by the overlapped formaldehyde and H_2_ in Figure 5c). Through spectral factorization, four alignment modes between ***P***_ini_ and ***P***_des_ can be determined (please refer to the detailed structures in Figure 5b), based on which a Gaussian-type attractive RTIP increasing over steps is defined as
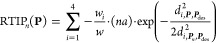
6where *n* denotes the searching step, *a* is the initial depth of the Gaussian function, and ***P***_*n*_ is the current configuration at step *n*.

In practical implementation, the RTIP is superimposed onto the real potential energy surface (PES) to enable pathway sampling, as depicted in Figure 5c. During the iterative optimization, the attractive RTIP Gaussian functions progressively increase, pushing the molecules toward closer for potential reactions, while the real PES applied on the molecules determines their ultimate reactivity. Throughout the optimization process, two crucial parameters are monitored at every iteration to ascertain the cutoff point. The first parameter, denoted as (*E*_*n*_ – *E*_ini_), is the energy difference between the current configuration (at step *n*) and the initial configuration on the real PES. This parameter must remain below the predefined threshold *E*_climb_ (115 kcal mol^–1^ in this work), as we aim to explore the low-energy reactions with *E*_a_ < *E*_climb_. Otherwise, it suggests that the molecules are either unreactive or misaligned for the potential reaction, resulting in a failed pathway sampling. The second parameter, represented as (*E*_max_ – *E*_*n*_), corresponds to the energy difference between the maximum-energy configuration (within the present pathway sampling) and the current configuration (at step *n*) on the real PES. When this parameter surpasses the specified threshold *E*_drop_ (12.5 kcal mol^–1^ in this work), it signifies the cleavage of preexisting chemical bonds and the subsequent formation of new bonds. Since the barrier has been overcome, it is imperative to promptly eliminate the RTIP, which is destined for the virtual ***P***_des_. Thereafter, a local optimization is carried on based on the real PES, driving the current configuration ***P***_*n*_ toward the desired products ***P***_pro_. In summary, the predefined thresholds *E*_climb_ and *E*_drop_ serve as the convergence criteria for the failed and successful pathway sampling, respectively: the former specifies the highest barrier in the search, while the latter refers to the release of energy for confirming the formation of new chemical bonds.

Throughout the pathway searching, the only prior assumption is that the selected molecules may get close to react, based on which the Gaussian-type attractive RTIP is constructed. The products ***P***_pro_ are predicted by the codriving of RTIP and the real PES, as exemplified by the RTIP trajectories for the synthesis of formic acid and formamide in Supporting information. Without more prior knowledges (e.g., the positions of the active sites), the current version of RTIP is not applicable to intramolecular reactions. We will consider this issue in the future.

### Program Implementation

The RTIP method has been implemented using the Rust programming language, which is available online (https://github.com/MillenniumDream/RTIP). As the pathway samplings are independent from each other, they can expediently perform in parallel (for instance, we performed 32 pathway samplings for each selected set of reactants in this study to guarantee a thorough exploration). In addition, RTIP is already integrated with the CP2K software, allowing direct access to quantum chemistry methods such as *x*TB and DFT within CP2K for potential energy surface calculations. Notably, we can also utilize CP2K’s QM/MM functionality to handle chemical reactions in solvent or other molecular environments. We will systematically test this feature in our future work.

There are still some shortcomings in the current version of RTIP. For example, the study did not precisely account for stoichiometric ratios, and the molecular reserve bank for reactants was selected based on human experience. In the future, we will introduce certain criteria or machine learning methods to automatedly consider these issues. We aim to achieve these goals in our upcoming work.

## Computational Details

In this study, both GFN0-*x*TB and DFT calculations were performed using the CP2K 2022.1 package (https://www.cp2k.org/)^61^ with the Quickstep electronic structure module.^50^ The semiempirical GFN0-*x*TB model^47,48^ was combined with the RTIP method to enable efficient pathway sampling. Afterward, the structures, energetics, and thermodynamic properties of the low-energy reactions were further refined by DFT calculations within the Gaussian and plane waves framework, i.e., TZVP basis set^51^ for the Gaussian functions along with a K mesh of 1 × 1 × 1 for the plane waves. Besides, the ωB97M-V functionals^49^ combined with VV10 nonlocal correlation^62^ was employed to ensure the high precision DFT computations. The detailed processes are elaborated below: (i) the reaction pathway was first optimized using the climbing image nudged elastic band (CI-NEB) method;^63^ (ii) subsequently, the TS structure was accurately located by the dimer method, with inspection of the imaginary vibration mode;^64^ (iii) an extrapolation optimization was further performed to obtain the stable reactant complex and product complex, allowing for the calculation of reaction energy and barrier; (iv) finally, the vibration frequencies and modes of the reactant complex, product complex, and TS structure were calculated using the finite difference method, with the corresponding Gibbs free energies derived by the Shermo program.^65^ The convergence criteria for the optimization were set as follows: the maximum force was set at 0.0002 hartree/bohr, the root-mean-square (RMS) force at 0.0001 hartree/bohr, the maximum geometry change at 0.002 bohr, and the RMS geometry change at 0.001 bohr.

## Data Availability

The data supporting this article have been included as part of the Supporting information.

## Supporting Information Available

Proof of non-negativity, symmetry, and triangle inequality for the metric; proof of roto-translational invariance of RTIP; supporting synthetic pathways for glycine, serine, and alanine; detailed information of the elementary reactions in prebiotic chemistry; coordinates of the TS structures in the main text (PDF)
RTIP trajectory for the synthesis of formic acid (R1) (MP4)
RTIP trajectory for the synthesis of formamide (R2) (MP4)
