# Supplementary material for: Discovery of New Synthetic Routes of Amino Acids in Prebiotic Chemistry
Source: JACS Au. 2024 Nov 18;4(12):4757–68. doi: 10.1021/jacsau.4c00685 (PMC11672127; doi:10.1021/jacsau.4c00685)
Supplement: Supplementary file 1 — au4c00685_si_001.pdf [file au4c00685_si_001.pdf]

Supporting Information for

Discovery of New Synthetic Routes of Amino Acids in Prebiotic Chemistry

Xiao-Tian Li[1*], Sixuan Mi[2], Yuzhi Xu[3], Bo-Wen Li[2], Tong Zhu[2-3,6*], John Z. H. Zhang[1-5*]

[1]Faculty of Synthetic Biology and Institute of Synthetic Biology, Shenzhen Institute of Advanced Technology, Chinese Academy of Sciences, Shenzhen 518055, China.

[2]Shanghai Engineering Research Center of Molecular Therapeutics & New Drug Development, School of Chemistry and Molecular Engineering, East China Normal University, Shanghai 200062, China.

[3]NYU-ECNU Center for Computational Chemistry at NYU Shanghai, Shanghai 200062, China.

[4]Department of Chemistry, New York University, New York 10003, United States.

[5]Collaborative Innovation Center of Extreme Optics, Shanxi University, Taiyuan, Shanxi 030006, China.

[6]Shanghai Innovation Institute, Shanghai, 200003 China.

Proof of non-negativity, symmetry, and triangle inequality for the metric

$$d_{\mathbf{P}_1,\mathbf{P}_2} := \min_{\mathbf{S}} \|\mathbf{P}_1 - \mathbf{S}\mathbf{P}_2\| \tag{1}$$

Proof:

Non-negativity. The metric is formulated as a Frobenius norm, which ensures its Non-negativity.

Symmetry.

$$d_{\mathbf{P}_1,\mathbf{P}_2} = \min_{\mathbf{S}} \|\mathbf{P}_1 - \mathbf{S}\mathbf{P}_2\| = \|\mathbf{P}_1 - \mathbf{S}_{1,2}\mathbf{P}_2\| = \left\|\mathbf{S}_{1,2}^{-1}\mathbf{P}_1 - \mathbf{P}_2\right\| = \left\|\mathbf{P}_2 - \mathbf{S}_{1,2}^{-1}\mathbf{P}_1\right\| \geq \min_{\mathbf{S}} \|\mathbf{P}_2 - \mathbf{S}\mathbf{P}_1\| = d_{\mathbf{P}_2,\mathbf{P}_1} \tag{2}$$

where $\mathbf{S}_{1,2}$ is the optimal roto-translation operation that makes $\|\mathbf{P}_1 - \mathbf{S}\mathbf{P}_2\|$ minimum, and $\mathbf{S}_{1,2}^{-1}$ is the inverse operation of $\mathbf{S}_{1,2}$ (It is noteworthy that **S** is a faithful representation of the SE(3) group). Likewise, $d_{\mathbf{P}_2,\mathbf{P}_1} \geq d_{\mathbf{P}_1,\mathbf{P}_2}$. Therefore, $d_{\mathbf{P}_1,\mathbf{P}_2} = d_{\mathbf{P}_2,\mathbf{P}_1}$.

Triangle inequality.

$$d_{\mathbf{P}_1,\mathbf{P}_2} + d_{\mathbf{P}_2,\mathbf{P}_3} = \min_{\mathbf{S}} \|\mathbf{P}_1 - \mathbf{S}\mathbf{P}_2\| + \min_{\mathbf{S}} \|\mathbf{P}_2 - \mathbf{S}\mathbf{P}_3\| = \|\mathbf{P}_1 - \mathbf{S}_{1,2}\mathbf{P}_2\| + \|\mathbf{P}_2 - \mathbf{S}_{2,3}\mathbf{P}_3\| = \|\mathbf{P}_1 - \mathbf{S}_{1,2}\mathbf{P}_2\| + \|\mathbf{S}_{1,2}\mathbf{P}_2 - \mathbf{S}_{1,2}\mathbf{S}_{2,3}\text{[illegible]} \tag{3}$$

Proof of roto-translational invariance of RTIP

Proof:

RTIP is formulated with the aforementioned metric in a combination manner

$$\mathrm{RTIP}(\mathbf{P}) := \sum_{i=1}^{n} f\left(d_{\mathbf{P},\mathbf{P}_i}\right) \tag{4}$$

where $\mathbf{P}_i$ represents a series of distinct structures within the configuration space of a molecular system. The mathematical formulation delineating the roto-translational invariance of RTIP is

$$\sum_{i=1}^{n} f\left(d_{\mathbf{P},\mathbf{P}_i}\right) = \sum_{i=1}^{n} f\left(d_{\mathbf{S}_0\mathbf{P},\mathbf{P}_i}\right) \tag{5}$$

where $\mathbf{S}_0$ denotes an arbitrary roto-translation operation acting on configuration variable $\mathbf{P}$. Without loss of generality, we only need to prove that $d_{\mathbf{P},\mathbf{P}_i} = d_{\mathbf{S}_0\mathbf{P},\mathbf{P}_i}$, as presented below.

$$d_{\mathbf{P},\mathbf{P}_i} = \min_{\mathbf{S}} \|\mathbf{P} - \mathbf{S}\mathbf{P}_i\| = \|\mathbf{P} - \mathbf{S}_i\mathbf{P}_i\| = \left\|\mathbf{P} - \mathbf{S}_i\left(\mathbf{S}_0^{-1}\mathbf{S}_0\right)\mathbf{P}_i\right\| = \left\|\mathbf{P} - \left(\mathbf{S}_i\mathbf{S}_0^{-1}\right)\mathbf{S}_0\mathbf{P}_i\right\| \geq \min_{\mathbf{S}} \|\mathbf{P} - \mathbf{S}\mathbf{S}_0\mathbf{P}_i\| = d_{\mathbf{P},\mathbf{S}_0\mathbf{P}_i} \tag{6}$$

$$d_{\mathbf{P},\mathbf{S}_0\mathbf{P}_i} = \min_{\mathbf{S}} \|\mathbf{P} - \mathbf{S}\mathbf{S}_0\mathbf{P}_i\| = \|\mathbf{P} - \mathbf{S}_i'\mathbf{S}_0\mathbf{P}_i\| = \left\|\mathbf{P} - \left(\mathbf{S}_i'\mathbf{S}_0\right)\mathbf{P}_i\right\| \geq \min_{\mathbf{S}} \|\mathbf{P} - \mathbf{S}\mathbf{P}_i\| = d_{\mathbf{P},\mathbf{P}_i} \tag{7}$$

where $\mathbf{S}_i$ and $\mathbf{S}_i'$ are the optimal roto-translation operations that make $\|\mathbf{P} - \mathbf{S}\mathbf{P}_i\|$ and $\|\mathbf{P} - \mathbf{S}\mathbf{S}_0\mathbf{P}_i\|$ minimum, respectively. Therefore, $d_{\mathbf{P},\mathbf{P}_i} = d_{\mathbf{S}_0\mathbf{P},\mathbf{P}_i}$.

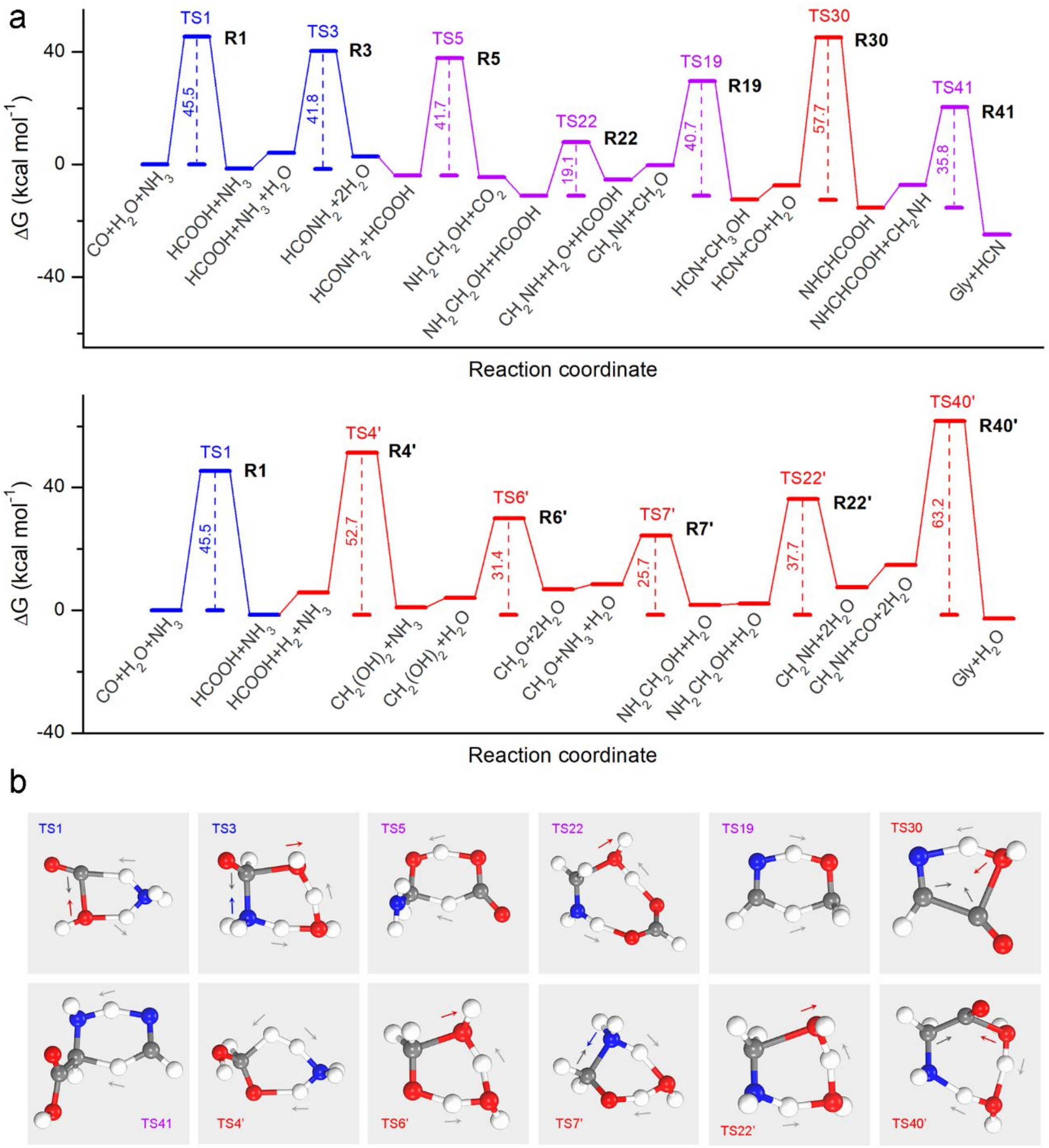

Figure S1. Supplementary synthetic pathways of glycine. (**a**) Gibbs free energy profile (at 298 K and 1 atm) for the competing synthetic pathways of glycine, as determined from the reaction network. For consistency, each elementary step is assigned a unique index that precisely follows the notation employed in the reaction network, while the apostrophe indicates a same type reaction, as summarized in Table S1. The color schemes are as follows: blue denotes previously documented reactions; purple indicates the newly identified reactions by RTIP; while red highlights the rate-determining steps for amino acid synthesis. (**b**) TS structures selected from **a** for illustration of the synthetic pathways. The arrows indicate the vibrational directionality for the atoms involved in bond cleavage and reformation within the imaginary normal mode at TS. Color code: gray ball for C, blue ball for N, red ball for O, white ball for H.

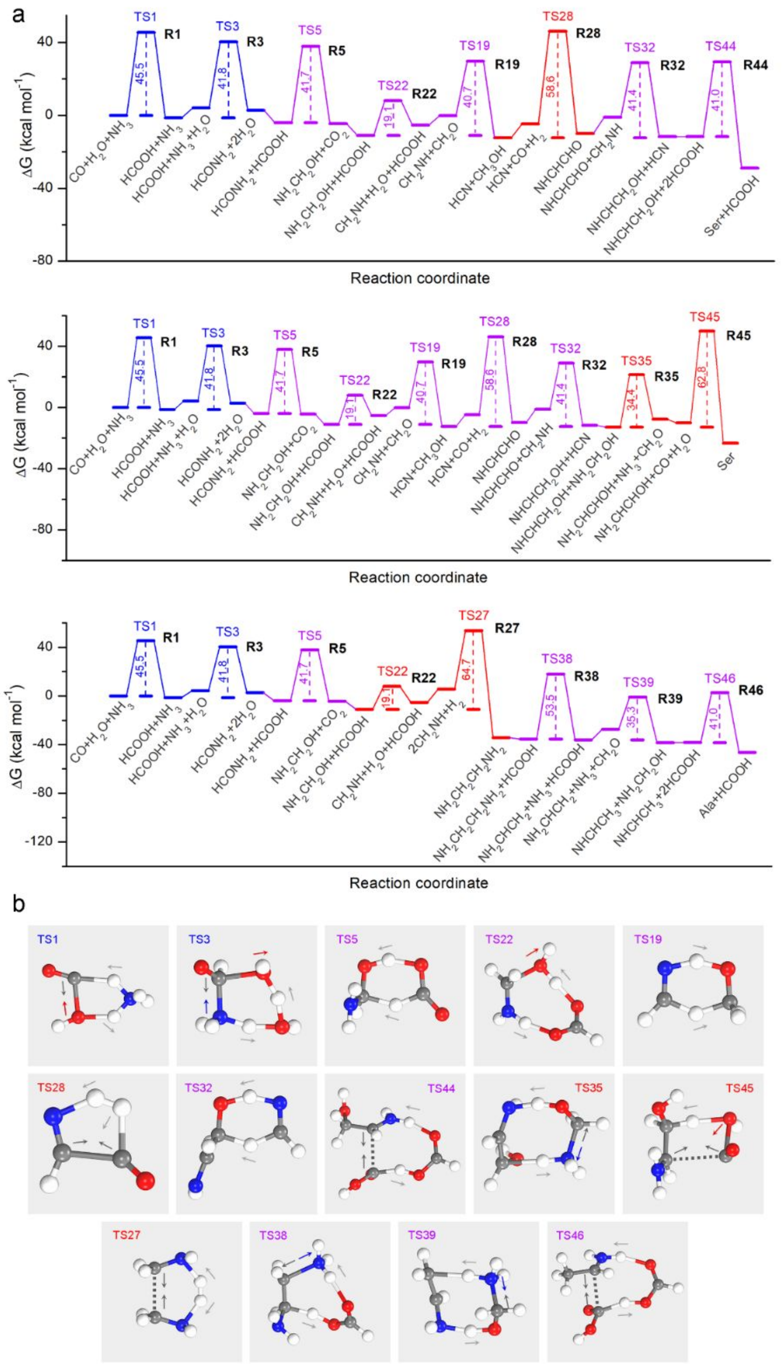

Figure S2. Supplementary synthetic pathways of serine and alanine. (**a**) Gibbs free energy profile (at 298 K and 1 atm) for the competing synthetic pathways of serine and alanine, as determined from the reaction network. For consistency, each elementary step is assigned a unique index that precisely follows the notation employed in the reaction network. The color schemes are as follows: blue denotes previously documented reactions; purple indicates newly identified reactions by RTIP; while red highlights the rate-determining steps for amino acid synthesis. (**b**) TS structures selected from **a** for illustration of the synthetic pathways. The arrows indicate the vibrational directionality for the atoms involved in bond cleavage and reformation within the imaginary normal mode at TS. Color code: gray ball for C, blue ball for N, red ball for O, white ball for H.

Table S1. Labeled index, reaction equation, catalyst (if exist), activation energy (E_a), reaction energy (ΔE), activation free energy (G_a), and reaction free energy (ΔG) of the elementary reactions in prebiotic chemistry.

Index*	Reaction equation	Catalyst	E_a (kcal mol^{-1})	ΔE (kcal mol^{-1})	G_a (kcal mol^{-1})	ΔG (kcal mol^{-1})
**1**	**$CO + H_2O \rightarrow HCOOH$**	**NH_3**	**41.3**	**-6.3**	**45.5**	**-1.4**
	$CO + H_2O \rightarrow HCOOH$	H_2O	45.7	-7.0		
**2**	**$CO + NH_3 \rightarrow HCONH_2$**	**H_2O**	**47.5**	**-11.4**	**49.5**	**-6.7**
	$CO + NH_3 \rightarrow HCONH_2$	NH_3	52.8	-13.3		
**3**	**$HCOOH + NH_3 \rightarrow HCONH_2 + H_2O$**	**H_2O**	**36.0**	**-1.5**	**36.2**	**-1.4**
	$HCOOH + NH_3 \rightarrow HCONH_2 + H_2O$	NH_3	38.0	-6.3		
**4**	**$2HCOOH \rightarrow CH_2(OH)_2 + CO_2$**		**45.0**	**-0.0**	**42.6**	**-1.1**
	$HCOOH + H_2 \rightarrow CH_2(OH)_2$	NH_3	42.6	-13.0	45.5	-4.8
	$HCOOH + CH_2NH \rightarrow CH_2(OH)_2 + HCN$		54.5	17.8	51.3	16.0
	$HCOOH + NH_3 + CH_2O \rightarrow CH_2(OH)_2 + HCONH_2$		44.2	-12.2		
	$HCOOH + H_2 \rightarrow CH_2(OH)_2$	H_2O	53.5	-2.7		
	$HCOOH + CH_2O \rightarrow CH_2(OH)_2 + CO$		55.7	6.7		
	$HCOOH + HCONH_2 \rightarrow CH_2(OH)_2 + HNCO$		57.6	20.6		
	$HCOOH + H_2 \rightarrow CH_2(OH)_2$		72.1	-5.7		
**5**	**$HCONH_2 + HCOOH \rightarrow NH_2CH_2OH + CO_2$**		**44.2**	**-0.6**	**41.7**	**-0.5**
	$HCONH_2 + H_2 \rightarrow NH_2CH_2OH$	NH_3	47.2	-10.0	49.7	-2.3
	$HCONH_2 + CH_2NH \rightarrow NH_2CH_2OH + HCN$		53.3	16.6	50.7	14.9
	$HCONH_2 + H_2 \rightarrow NH_2CH_2OH$		69.4	-2.6	69.9	4.4
	$HCONH_2 + NH_3 + CH_2O \rightarrow NH_2CH_2OH + HCONH_2$		47.0	-7.5		
	$HCONH_2 + H_2 \rightarrow NH_2CH_2OH$	H_2O	50.8	-1.2		
	$HCONH_2 + CH_2O \rightarrow NH_2CH_2OH + CO$		53.6	2.1		
	$2HCONH_2 \rightarrow NH_2CH_2OH + HNCO$		58.1	15.4		
**6**	**$CH_2O + H_2O \rightarrow CH_2(OH)_2$**	**HCOOH**	**8.9**	**-12.4**	**9.3**	**-7.7**
	$CH_2O + H_2O \rightarrow CH_2(OH)_2$	H_2O	22.0	-7.8	23.2	-2.7
	$CH_2O + H_2O \rightarrow CH_2(OH)_2$	NH_3	21.5	-11.2	23.7	-7.2
**7**	**$CH_2O + NH_3 \rightarrow NH_2CH_2OH$**	**HCOOH**	**2.9**	**-14.0**	**4.0**	**-10.2**
	$CH_2O + NH_3 \rightarrow NH_2CH_2OH$	H_2O	13.2	-12.1	15.8	-6.7
	$CH_2O + NH_3 \rightarrow NH_2CH_2OH$	NH_3	19.7	-12.3	21.7	-7.3
**8**	**$CO + HCOOH \rightarrow CH_2O + CO_2$**		**53.1**	**-4.3**	**52.0**	**-4.7**
**9**	**$H_2 + CO \rightarrow CH_2O$**	**H_2**	**58.9**	**-5.4**	**64.5**	**2.9**
	$H_2 + CO \rightarrow CH_2O$	NH_3	58.9	-5.7		
	$H_2 + CO \rightarrow CH_2O$	H_2O	65.1	-5.3		
**10**	**$2CH_2O + H_2O \rightarrow HCOOH + CH_3OH$**		**41.2**	**-34.1**	**42.1**	**-28.9**
	$CH_2O + H_2O + HCONH_2 \rightarrow HCOOH + NH_2CH_2OH$		44.1	-13.9		
	$CH_2O + H_2O + HCN \rightarrow HCOOH + CH_2NH$		54.1	-16.2		
**11**	**$2CH_2O + NH_3 \rightarrow HCONH_2 + CH_3OH$**		**33.3**	**-31.7**	**36.8**	**-28.0**
	$CH_2O + NH_3 + CH_2NH \rightarrow HCONH_2 + CH_3NH_2$		40.5	-33.8		
	$CH_2O + NH_3 + HCN \rightarrow HCONH_2 + CH_2NH$		47.5	-22.8		
**12**	**$HCOOH + CH_2NH \rightarrow CO_2 + CH_3NH_2$**		**32.0**	**-25.9**	**31.4**	**-25.3**
	$HCOOH + CH_2O \rightarrow CO_2 + CH_3OH$		32.3	-24.9	31.4	-23.6
	$HCOOH + HCN \rightarrow CO_2 + CH_2NH$		43.8	-13.7	42.2	-12.6
**13**	**$HCONH_2 + CH_2O \rightarrow HNCO + CH_3OH$**		**41.5**	**-7.2**	**40.7**	**-6.1**
	$HCONH_2 + CH_2NH \rightarrow HNCO + CH_3NH_2$		46.6	-4.7		
	$HCONH_2 + HCN \rightarrow HNCO + CH_2NH$		55.7	8.6		
**14**	**$H_2CO_3 \rightarrow CO_2 + H_2O$**	**HCOOH**	**17.2**	**-10.3**	**13.7**	**-14.3**
	$H_2CO_3 \rightarrow CO_2 + H_2O$	NH_3	22.5	-1.1	20.3	-5.5
	$H_2CO_3 \rightarrow CO_2 + H_2O$	H_2O	25.4	-2.9	22.7	-6.8
**15**	**$HCOOH + H_2O + CH_2O \rightarrow H_2CO_3 + CH_3OH$**		**55.3**	**-20.9**	**57.7**	**-14.4**
**16**	**$C_2H_2O_3 \rightarrow CO + HCOOH$**		**30.9**	**-3.2**	**27.3**	**-7.7**
**17**	**$HNCO + NH_3 \rightarrow CO(NH_2)_2$**	**HCOOH**	**23.3**	**-14.1**	**24.6**	**-9.2**
	$HNCO + NH_3 \rightarrow CO(NH_2)_2$	NH_3	35.4	-15.2	40.6	-9.0
	$HNCO + NH_3 \rightarrow CO(NH_2)_2$	H_2O	40.7	-16.4	42.0	-11.8
**18**	**$NCOH \rightarrow HNCO$**	**HCOOH**	**5.6**	**-17.2**	**3.3**	**-18.4**
	$NCOH + NH_2CH_2OH \rightarrow HNCO + CH_2O + NH_3$		8.1	-2.9		
	$NCOH + NH_2CH_2OH \rightarrow HNCO + CH_2NH + H_2O$		10.1	-7.7		
	$NCOH + CH_2(OH)_2 \rightarrow HNCO + CH_2O + H_2O$		13.1	-6.6		
**19**	**$CH_2O + CH_2NH \rightarrow CH_3OH + HCN$**		**31.0**	**-12.1**	**29.9**	**-12.2**
	$2CH_2O \rightarrow CH_3OH + CO$		33.8	-23.2		
**20**	**$H_2 + CH_2O \rightarrow CH_3OH$**	**NH_3**	**31.1**	**-31.6**	**33.9**	**-24.8**
	$H_2 + CH_2O \rightarrow CH_3OH$	H_2O	37.3	-27.7		
	$H_2 + CH_2O \rightarrow CH_3OH$	H_2	39.8	-29.3		
	$H_2 + CH_2O \rightarrow CH_3OH$		66.2	-28.7		
**21**	**$CH_3OH + H_2 \rightarrow CH_4 + H_2O$**	**HCOOH**	**66.6**	**-30.3**	**67.3**	**-28.3**

	$CH_3OH + HNCO + H_2 \rightarrow CH_4 + NCOH + H_2O$		73.8	-7.8		
	$CH_3OH + HCOOH \rightarrow CH_4 + CO_2 + H_2O$		85.3	-24.5		
**22**	**$CH_2NH + H_2O \rightarrow NH_2CH_2OH$**	**HCOOH**	**11.5**	**-9.8**	**13.3**	**-5.8**
	$CH_2NH + H_2O \rightarrow NH_2CH_2OH$	H_2O	29.5	-8.9	28.7	-5.4
	$CH_2NH + H_2O \rightarrow NH_2CH_2OH$	NH_3	33.8	-9.1	34.0	-5.8
	$HCN + CH_2NH \rightarrow CH_2NH + HCN$		41.4	0.0	41.2	0.0
	$HCN + CH_2O \rightarrow CH_2NH + CO$		42.9	-11.9		
**23**	**$H_2 + HCN \rightarrow CH_2NH$**	**NH_3**	**48.0**	**-15.2**	**53.0**	**-6.1**
	$H_2 + HCN \rightarrow CH_2NH$	H_2	47.4	-18.1	53.8	-10.4
	$H_2 + HCN \rightarrow CH_2NH$	H_2O	50.5	-17.7		
	$H_2 + HCN \rightarrow CH_2NH$		85.2	-18.0		
	$2CH_2NH \rightarrow CH_3NH_2 + HCN$		33.5	-12.9	31.8	-13.7
	$CH_2NH + CH_2O \rightarrow CH_3NH_2 + CO$		39.5	-24.6		
**24**	**$H_2 + CH_2NH \rightarrow CH_3NH_2$**	**H_2**	**40.5**	**-33.7**	**44.3**	**-28.0**
	$H_2 + CH_2NH \rightarrow CH_3NH_2$	NH_3	43.5	-32.0	46.2	-25.3
	$H_2 + CH_2NH \rightarrow CH_3NH_2$	H_2O	45.7	-28.6		
	$H_2 + CH_2NH \rightarrow CH_3NH_2$		76.0	-33.8		
**25**	**$CH_2NH + CO + H_2 \rightarrow NH_2CH_2CHO$**		**40.0**	**-31.8**	**44.6**	**-20.2**
	$CH_2NH + CO + HCOOH \rightarrow NH_2CH_2CHO + CO_2$		44.8	-27.5		
**26**	**$CH_2NH + CH_2O + H_2 \rightarrow NH_2CH_2CH_2OH$**		**44.6**	**-50.5**	**49.6**	**-37.4**
**27**	**$2CH_2NH + H_2 \rightarrow NH_2CH_2CH_2NH_2$**		**43.3**	**-52.8**	**47.9**	**-40.0**
**28**	**$HCN + CO + H_2 \rightarrow NHCHCHO$**		**46.1**	**-17.6**	**50.8**	**-5.3**
**29**	**$HCN + CH_2O + H_2 \rightarrow NHCHCH_2OH$**		**48.0**	**-38.7**	**55.0**	**-23.8**
**30**	**$HCN + CO + H_2O \rightarrow NHCHCOOH$**		**48.1**	**-18.1**	**52.6**	**-8.0**
	$HCN + HCOOH \rightarrow NHCHCOOH$	HCOOH	65.6	-7.0		
**31**	**$NHCHCHO + CH_2NH \rightarrow NH_2CH_2CHO + HCN$**		**30.6**	**-18.3**	**27.8**	**-18.3**
	$NHCHCHO + CH_3OH \rightarrow NH_2CH_2CHO + CH_2O$		34.1	-6.6	31.9	-7.1
	$NHCHCHO + HCOOH \rightarrow NH_2CH_2CHO + CO_2$		33.9	-27.6		
	$NHCHCHO + CH_2O \rightarrow NH_2CH_2CHO + CO$		37.6	-25.2		
**32**	**$NHCHCHO + CH_2NH \rightarrow NHCHCH_2OH + HCN$**		**32.0**	**-10.3**	**30.0**	**-10.6**
	$NHCHCHO + HCOOH \rightarrow NHCHCH_2OH + CO_2$		34.5	-23.1		
	$NHCHCHO + CH_2O \rightarrow NHCHCH_2OH + CO$		35.5	-18.8		
**33**	**$NH_2CH_2CHO + HCOOH \rightarrow NH_2CH_2CH_2OH + CO_2$**		**32.6**	**-21.9**	**32.2**	**-18.9**
	$NH_2CH_2CHO + CH_2NH \rightarrow NH_2CH_2CH_2OH + HCN$		34.7	-8.3		
	$NH_2CH_2CHO + CH_2O \rightarrow NH_2CH_2CH_2OH + CO$		37.0	-19.5		
**34**	**$NHCHCH_2OH + HCOOH \rightarrow NH_2CH_2CH_2OH + CO_2$**		**32.5**	**-22.5**	**30.9**	**-21.4**
	$NHCHCH_2OH + CH_3OH \rightarrow NH_2CH_2CH_2OH + CH_2O$		34.0	-3.6	32.2	-2.2
	$NHCHCH_2OH + CH_2NH \rightarrow NH_2CH_2CH_2OH + HCN$		38.2	-13.2		
	$NHCHCH_2OH + CH_2O \rightarrow NH_2CH_2CH_2OH + CO$		43.8	-23.8		
**35**	**$NH_2CHCHOH + CH_2O + NH_3 \rightarrow NHCHCH_2OH + NH_2CH_2OH$**		**25.1**	**-10.6**	**29.2**	**-5.2**
	$NH_2CHCHOH + CH_2O + H_2O \rightarrow NHCHCH_2OH + CH_2(OH)_2$		40.6	-8.6		
	$NH_2CHCHOH + CH_2NH + H_2O \rightarrow NHCHCH_2OH + NH_2CH_2OH$		41.1	-9.4		
**36**	**$NH_2CHCHOH + HCOOH \rightarrow NH_2CH_2CH_2OH + CO_2$**		**32.7**	**-25.5**	**33.8**	**-22.7**
	$NH_2CHCHOH + CH_2NH \rightarrow NH_2CH_2CH_2OH + HCN$		45.2	-14.2		
	$NH_2CHCHOH + CH_2O \rightarrow NH_2CH_2CH_2OH + CO$		49.8	-20.6		
**37**	**$NH_2CHCH_2 + H_2O \rightarrow NH_2CH_2CH_2OH$**	**HCOOH**	**56.0**	**-6.4**	**53.4**	**-2.1**
	$NH_2CHCH_2 + H_2O \rightarrow NH_2CH_2CH_2OH$	H_2O	63.5	0.8		
	$NH_2CHCH_2 + H_2O \rightarrow NH_2CH_2CH_2OH$	NH_3	68.3	1.7		
**38**	**$NH_2CHCH_2 + NH_3 \rightarrow NH_2CH_2CH_2NH_2$**	**HCOOH**	**56.6**	**-2.0**	**54.1**	**0.6**
	$NH_2CHCH_2 + NH_3 \rightarrow NH_2CH_2CH_2NH_2$	H_2O	57.5	-4.7		
	$NH_2CHCH_2 + NH_3 \rightarrow NH_2CH_2CH_2NH_2$	NH_3	65.8	-3.5		
**39**	**$NH_2CHCH_2 + CH_2O + NH_3 \rightarrow NHCHCH_3 + NH_2CH_2OH$**		**25.1**	**-14.8**	**26.5**	**-11.0**
	$NH_2CHCH_2 + CH_2O + H_2O \rightarrow NHCHCH_3 + CH_2(OH)_2$		35.8	-16.2		
	$NH_2CHCH_2 + CH_2NH + H_2O \rightarrow NHCHCH_3 + NH_2CH_2OH$		39.8	-11.9		
**40**	**$CH_2NH + HCOOH \rightarrow$ Glycine**	**HCOOH**	**43.1**	**-21.9**	**41.9**	**-18.5**
	$CH_2NH + CO + H_2O \rightarrow$ Glycine		43.7	-31.5	46.8	-21.6
	$CH_2NH + CO + H_2O \rightarrow$ Glycine	H_2O	44.3	-25.7	47.0	-17.5
	$CH_2NH + HCOOH \rightarrow$ Glycine	H_2O	51.8	-21.8		
	$CH_2NH + CO_2 + H_2 \rightarrow$ Glycine		73.5	-21.6		
**41**	**$NHCHCOOH + CH_2NH \rightarrow$ Glycine + HCN**		**30.2**	**-17.0**	**27.7**	**-17.6**
	$NHCHCOOH + HCOOH \rightarrow$ Glycine + CO_2		33.7	-30.6		
	$NHCHCOOH + CH_2O \rightarrow$ Glycine + CO		41.9	-21.1		
	$NHCHCOOH + HCONH_2 \rightarrow$ Glycine + HNCO		48.5	-12.9		

**42**	**Glycine + CH_2O → Serine**	**HCOOH**	**51.2**	**-13.3**	**47.9**	**-12.1**
**43**	**NHCHCOOH + CH_2O + H_2 → Serine**		**43.2**	**-47.8**	**47.9**	**-36.3**
**44**	**$NHCHCH_2OH$ + HCOOH → Serine**	**HCOOH**	**41.9**	**-21.5**	**40.9**	**-17.2**
	$NHCHCH_2OH$ + CO +H_2O → Serine		43.6	-32.8		
	$NHCHCH_2OH$ + HCOOH → Serine	HNCO	50.8	-15.3		
**45**	**$NH_2CHCHOH$ + CO + H_2O → Serine**		**58.1**	**-21.9**	**60.0**	**-13.3**
**46**	**$NHCHCH_3$ + HCOOH → Alanine**	**HCOOH**	**41.5**	**-13.2**	**40.8**	**-8.2**
	$NHCHCH_3$ + CO + H_2O → Alanine		43.3	-27.2		
	$NHCHCH_3$ + HCOOH → Alanine	HNCO	44.6	-18.3		
**47**	**NH_2CHCH_2 + CO + H_2O → Alanine**		**53.2**	**-32.5**	**54.1**	**-23.3**
	NHCHCOOH + CH_4 → Alanine	HCOOH	64.5	-19.7		

Coordinates of the TS Structures in the main text

9
TS of R1 in the main text

C	0.3782795223	1.1721504479	0.5564933343
O	1.1461744359	2.0444272600	0.6458149720
O	1.0996991551	-0.2740213881	-0.0208792241
H	-0.2675931583	-1.0664294693	-0.1954216979
H	2.0171229477	-0.0927612676	-0.2564121991
N	-1.2820895487	-0.8336170299	0.0350402593
H	-1.8590014106	-0.7754762753	-0.7956290559
H	-1.0337004219	0.1614532464	0.4291496200
H	-1.6918915215	-1.4527255241	0.7238439915

9
TS of R2 in the main text

C	0.0134329741	1.1490284292	0.0045028921
O	-0.4024919404	2.2170065136	-0.2151778316
O	1.1633482734	-1.0125599650	0.3414614778
H	0.9643935178	0.3476319707	0.2328511923
H	1.8415565113	-1.4134861571	-0.2065487575
N	-1.0559208149	-0.1594205634	-0.1463093352
H	-1.5329890343	-0.1575832642	-1.0413845148
H	-1.7389712546	-0.1679649136	0.6040103444
H	-0.2080222324	-0.9028780501	-0.0269424674

12
TS of R3 in the main text

N	-0.6985644097	0.5146585054	0.3724597842
H	-0.6982675704	0.9932894567	1.2674209897
H	-0.8997715484	1.2046618576	-0.3482987325
H	0.3323660187	-0.0833094540	0.1224974941
O	1.1201798437	-0.9432824259	-0.4266634058
H	0.0809614044	-1.4982678631	-0.6758627715
H	1.6645924427	-1.4662601671	0.1699535522
O	-1.1070983629	-1.7283801691	-0.6654669860
C	-1.8876151621	-0.4582095115	0.3332558884
O	-2.9000863644	-0.0546893400	-0.2026187697
H	-1.5591542679	-1.6290303481	-1.5118032872
H	-1.8612953238	-1.0680407008	1.2457787540

10
TS of R4 in the main text

O	-1.1642750668	0.2807000259	-0.7894193660
C	-0.7604891557	-0.6812768488	-0.0316322388

O	-1.1420585794	-0.7146417020	1.2531614290
H	-0.2974091895	0.9820301710	-0.9161116339
H	-0.6377227028	-1.6878218085	-0.4344185152
O	0.9061102669	1.4336244243	-0.8003550418
C	1.5221694631	0.5146301934	-0.2359856930
O	2.6094302371	0.2238551492	0.1411770383
H	-1.4700837760	0.1676123474	1.4871631601
H	0.6596835732	-0.4151186920	0.0143724815

11
TS of R5 in the main text

O	-0.9739535245	0.0893925167	-1.4869768798
C	-1.1575185685	0.3678632331	-0.2977592183
O	-2.0360855383	0.5159892944	0.4985112401
H	0.4396289345	0.1225795182	-1.6035301285
H	-0.0136024646	0.5303349549	0.2423052674
N	1.7159540848	-0.9022050377	0.7157441375
C	1.4184236280	0.2346916625	0.0378118849
O	1.4410236045	0.2011595004	-1.2709893513
H	1.6939255304	-0.8863117951	1.7212126325
H	1.4871234941	-1.7758369464	0.2684018983
H	1.7259570996	1.1553250390	0.5384632073

12
TS of R6 in the main text

O	-0.6310709332	0.9620609235	0.3515292023
H	-0.4059269212	1.2194830070	1.2565214319
H	0.2664481983	0.7701899702	-0.1432233923
O	1.5326168017	-1.8185459053	-0.1820578975
C	1.9443882335	-0.8203500439	-0.8534168895
O	1.4509885478	0.3182442504	-0.8945337575
H	0.5942533571	-1.6189518110	0.5228798169
H	2.8372462008	-1.0175584210	-1.4622056324
O	-0.3875141043	-1.3560968392	1.2012491501
C	-1.2122692278	-0.6986750000	0.4886709728
H	-2.1835312897	-0.4651906050	0.9237257159
H	-1.2261122531	-0.8547898257	-0.5946212906

13
TS of R7 in the main text

N	-0.5196322071	1.4216433492	0.1456641578
H	0.4526370031	1.1114324961	0.2313071478
H	-0.7478481992	2.0963953983	0.8674327862
H	-0.6780829508	1.8247148247	-0.7705562912
O	1.2133937633	-2.0269462591	-0.4692941644
C	2.0253659084	-1.2860725820	0.2272271057
O	1.8409353837	-0.1374503364	0.5862167436

H	0.3285024520	-1.5293605524	-0.7130048011
H	2.9481917975	-1.8222908349	0.4859209065
O	-0.9611224180	-0.8492776508	-0.9668407676
C	-1.3755810971	-0.3011492167	0.0794974434
H	-2.4018924477	0.0820588348	0.1184598751
H	-0.9758669880	-0.6046974707	1.0579698583

7
TS of R8 in the main text

O	0.1491421315	0.8031744998	0.9890112294
C	0.1124252238	1.0357146291	-0.2240453810
O	-0.2898144236	1.8127095405	-1.0296902138
H	0.8842403854	-0.3979234558	0.9363697084
H	0.7736977907	0.0254483386	-0.7869543966
O	1.9076453619	-1.9793928244	-0.4144820802
C	1.3643595702	-1.0933864877	0.0490287238

6
TS of R9 in the main text

C	0.0724435186	2.1254425369	-0.2272481635
O	0.3316672247	2.9125639589	0.5542710971
H	0.1791570281	0.4371678825	-1.4985980546
H	0.7265750143	1.1807030276	-1.1461320739
H	-0.8205274072	0.4770941238	-1.2072617453
H	-1.0380841085	1.2520444704	-0.6314232198

11
TS of R10 in the main text

O	0.2407880413	1.1143463534	0.1225455407
H	-0.8157052296	0.8237695992	0.2443612967
H	0.5580142397	1.3978833859	0.9938571110
O	1.6747287110	-0.7051785361	0.8816517458
C	1.0249964035	-0.3699442985	-0.0706959166
H	-0.0916844889	-0.9984259254	-0.2331319639
H	1.3662858318	-0.2289220290	-1.1037995360
O	-1.8787165042	0.0473676383	0.3497323098
C	-1.4858773162	-1.0772107719	-0.1050993593
H	-1.6483859863	-1.3248447601	-1.1659590912
H	-1.4682035720	-1.9461348658	0.5665193829

12
TS of R11 in the main text

N	0.2899885834	1.1180274564	0.0509518148
H	0.8189941850	1.6551358128	-0.6298364099
H	0.1912657872	1.6289889294	0.9224854878

H	-0.6935603357	0.8602189729	-0.3481676470
O	1.7989487520	-0.5031790153	-0.7441599485
C	1.0494631607	-0.2408641096	0.1980467142
H	1.3503143359	-0.3257488063	1.2600881921
H	-0.0283490723	-0.9693254436	0.2453902226
O	-1.7982280956	-0.0705421898	-0.6652511964
C	-1.3629678376	-1.1734227683	-0.2185142950
H	-1.0410848239	-1.9583624304	-0.9199032579
H	-1.7286158091	-1.5503682682	0.7501425131

10

TS of R12 in the main text

O	-1.2756520444	-0.5848764847	0.8302436441
C	-1.4908936291	-0.0020035389	-0.2547553488
O	-2.4583811231	0.2504652179	-0.9273599401
H	0.2046046546	-0.6450271999	1.0837261597
H	-0.4541724426	0.3978567862	-0.7430191404
C	1.2169374832	-0.0023663947	-0.4681847138
N	1.1504813905	-0.1688737936	0.8223086974
H	1.7148024329	0.8609527287	-0.8927756817
H	1.0321183325	-0.8518966827	-1.1119276689
H	1.3751549455	0.6407693617	1.3867439925

10

TS of R13 in the main text

C	-0.0129581543	1.3677443219	0.7255025357
O	-0.5265690765	1.8474570395	-0.3618266304
H	1.0706808680	1.1835702592	0.7539795179
H	-0.3912887320	0.1277833182	0.8340875844
N	-1.4755243745	-0.3051413359	-0.8392228351
C	-1.0622387739	-0.9238084737	0.1912320358
O	-0.9931472493	-1.9240504126	0.8178185010
H	-1.1193630201	0.8394049775	-0.8419865625
H	-1.9813878794	-0.7876299736	-1.5624179471
H	-0.4182156880	1.7114568694	1.6874750903

11

TS of R14 in the main text

O	-0.3009430915	0.7876473288	0.7006576634
H	-0.3255834891	1.3665352122	-0.0750643120
H	0.7733151519	0.2289251422	0.7088127730
O	1.1548351811	-1.3616212984	-1.2304210557
C	2.0101616306	-1.0685172845	-0.3460840425
O	1.8286135756	-0.3457239877	0.6618773421
H	0.0937183365	-1.0732854613	-0.9981772755
H	3.0069028630	-1.4908892253	-0.4847594690
C	-1.3844166695	-0.3459807393	0.4400414364

O	-2.1535505219	-0.4594069698	1.3259516068
O	-1.1500746668	-0.8696641269	-0.6701120869

12
TS of R15 in the main text

O	0.0921092770	0.7232253864	-0.3150801266
H	0.3854237344	1.2203332316	0.4616184125
H	-0.9908009020	0.4219926456	-0.1210599598
O	1.3999317400	-0.6503913108	1.0502456890
C	0.8971069508	-0.6934150212	-0.2410246182
O	1.5431805493	-0.9762450487	-1.2162894452
H	2.3106143699	-0.9656876424	0.9687270038
H	-0.1528445598	-1.3808455203	-0.1006608942
C	-1.5657127395	-1.4765426324	-0.0533499913
O	-1.9651530600	-0.3056862325	0.2535132258
H	-1.6210940448	-2.2667141051	0.7049065105
H	-1.6129076954	-1.8231785702	-1.0950233164

7
TS of R16 in the main text

C	-0.5514910977	1.1647491556	-0.0742335918
O	-0.0431511305	2.0561495921	0.4162135679
O	-1.6293711646	-0.9020299824	-0.8751352887
C	-1.2339064025	-1.1750000293	0.2848297722
O	-0.6222059805	-0.3438635272	1.0244550597
H	-1.1510755345	0.3732069146	-0.8392972284
H	-1.4205551397	-2.1787232635	0.6909722991

13
TS of R17 in the main text

N	-0.5913545992	1.1611284645	0.2074411171
H	0.4547071505	0.8655477113	0.1649708040
H	-0.8255930632	1.7576573039	-0.5811158140
H	-0.8382799568	1.6182242656	1.0811939417
O	1.2091119650	-2.0445832653	-0.1852439904
C	2.0252493717	-1.1042055786	0.0407851607
O	1.7910585594	0.1227029630	0.0666733829
H	0.0714003194	-1.6334048295	-0.5249429893
H	3.0603740594	-1.4240104076	0.2304408789
N	-0.9086478265	-0.9485259687	-0.9006223733
C	-1.2919379860	-0.2410901148	0.1581809850
O	-1.9347051993	-0.5042589186	1.1306690968
H	-0.4823827944	-0.3601816251	-1.6104302001

9

TS of R18 in the main text

O	0.7190574982	-0.8808165699	-2.3941762017
C	1.4378478344	-0.0892058645	-3.0590519590
O	2.3641166651	0.6748011478	-2.6657000585
H	0.8430482932	-1.0015650917	-1.2957563515
H	1.2343385799	-0.0562197527	-4.1278458324
N	2.8451738653	0.5674075313	-0.1258584914
C	2.0045107775	-0.2350536519	0.0446984011
O	1.0925922731	-1.0608913491	0.0240345117
H	2.6044848734	0.6740107608	-1.6159301882

9

TS of R19 in the main text

O	1.4382355534	-1.1670882978	-0.8916072947
C	1.6006568772	-0.6845875668	0.2884299446
H	1.9577255477	0.3481010312	0.3836646091
H	1.9333917299	-1.3610234232	1.0847177734
C	-0.9207116537	-0.6295352967	0.3022842403
N	-0.9595268035	-1.0762190528	-0.8083647330
H	0.3261799501	-0.4742576247	0.7565384210
H	-1.6088919723	-0.3053111296	1.0694739799
H	0.2331099012	-1.2246231397	-1.1104334405

10

TS of R20 in the main text

H	0.6249232425	-0.6428638049	0.2828236999
H	-0.2607299287	-0.3163589942	0.6001655657
N	1.5208027177	-1.3560877649	-0.3629250736
H	0.7800696651	-1.9205689793	-0.8228284490
H	2.0714083510	-0.8318336008	-1.0277581664
H	2.1099741157	-1.9174987511	0.2352218784
O	-0.9448488825	-1.9922744979	-0.8148532085
C	-1.3780909304	-1.1140353115	-0.0250823856
H	-1.8539292606	-0.2001304472	-0.4179228477
H	-1.7701562599	-1.4016317282	0.9644861768

13

TS of R21 in the main text

H	-1.1042760693	-2.9488865085	0.3143354188
H	-1.9166674831	-2.7341458665	0.4256668654
O	1.2290665300	-1.1946358983	-0.6787581781
C	1.3146215661	-2.4007925531	-0.3476741078
O	0.4132152172	-3.1772791610	0.0650408429
H	0.1283870365	-0.0708434421	-0.8179885645
H	2.3207484739	-2.8513066782	-0.4251786035
H	-2.5514574729	-0.8402321396	0.0441418696
C	-1.5342744155	-1.1872658676	-0.0778283724

H	-1.2291712023	-1.6145833721	-1.0135771503
H	-0.8402603824	-1.1439815232	0.7386402592
O	-0.7430714437	0.4550851100	-0.7814779864
H	-0.6206758746	1.1455243003	-0.1191924129

13
TS of R22 in the main text

O	-0.6393716488	0.7669827120	-0.8950384656
H	-0.4984566765	1.6991410676	-0.6894885302
H	0.3122632285	0.3121292032	-0.8515387291
O	1.4550194707	-1.4247409666	1.1460542362
C	2.0874874627	-0.9531421495	0.1749364309
O	1.6329583588	-0.2742152148	-0.7822251458
H	-0.0759994109	-1.5700929118	0.8396719372
H	3.1724427238	-1.1569407271	0.1427957049
C	-1.5808702905	-0.2707128779	0.4793884871
N	-1.0690818212	-1.4642423113	0.4624407176
H	-2.5438475102	-0.0942733780	0.0247176133
H	-1.2614744290	0.3942007271	1.2703757376
H	-1.4033551776	-2.1229131029	-0.2218882541

9
TS of R23 in the main text

H	0.1307950927	0.0178988896	-0.2542445082
H	-0.9624398201	0.0653757291	-0.1579939606
N	1.2250617555	-0.5301861591	-0.3373464951
H	0.7927829999	-1.4889665227	-0.2806404200
H	1.8328263166	-0.3355489207	0.4447168431
H	1.6923085415	-0.3699158349	-1.2178762166
H	-2.5666657283	-1.0884696103	0.0188558855
C	-1.5207439116	-1.3493602255	-0.0742709439
N	-0.7757997862	-2.2682026554	-0.1241361141

9
TS of R24 in the main text

H	-1.7258280767	-0.0244924246	0.4102587086
H	-0.8513180986	0.0153590925	0.3836188015
H	-0.4029160657	-1.4724191810	-0.1828823839
H	0.0019495439	-0.6662248953	0.0175602582
C	-2.4928247728	-1.3105948184	-0.0175104308
N	-1.5541682811	-2.1099370120	-0.4972037937
H	-3.1708510136	-0.7708156144	-0.6746004780
H	-2.8474928043	-1.4932677554	0.9924531719
H	-1.4902026510	-2.0125018913	-1.5097815838

9
TS of R25 in the main text

C	-0.5963293721	1.0129471585	-0.0915859717
O	-0.2125706237	1.9161189740	-0.6689530130
H	0.2982845712	-0.2935519178	0.3827723032
H	-0.2635597159	-1.0204427997	0.4981082304
C	-2.1978138695	-0.2982461227	0.1250196816
N	-1.5419135585	-1.4365109544	0.2525208410
H	-2.6643291053	-0.0003478848	-0.8111608382
H	-2.6033102226	0.1581195565	1.0219582395
H	-1.3855816736	-1.8610856996	-0.6611381928

11
TS of R26 in the main text

H	-0.6088455376	0.6624035677	-0.0224541831
H	0.2691557926	0.7412753085	-0.0851555778
O	1.4302392129	0.0238802648	0.1412104159
C	1.2277599301	-1.2064830740	0.2749184923
H	1.2694879834	-1.6512154438	1.2725656451
H	1.3937874119	-1.8716248878	-0.5775608772
C	-0.9512561888	-1.5493416149	0.2414963878
N	-1.5149678496	-0.4136161302	-0.1111552666
H	-0.8049178988	-2.3439673042	-0.4815544445
H	-1.0138600895	-1.8248762808	1.2874353419
H	-1.6006419365	-0.3534244853	-1.1257729138

12
TS of R27 in the main text

H	-0.2334789479	0.0905196717	-0.8456356100
H	0.6534473518	0.0341426284	-0.7723403468
C	1.1317181496	-1.6307958189	0.6839780110
N	1.6602274042	-0.7919716099	-0.1764073253
H	1.0478060374	-2.6903048259	0.4624992183
H	1.1118815615	-1.3392242033	1.7278076117
H	1.8488451615	-1.2614595279	-1.0613984486
C	-1.1410832555	-1.3748528653	0.5896616108
N	-1.4156666455	-0.5695315143	-0.4073889299
H	-1.2257523992	-2.4519811755	0.4941427817
H	-1.1719549020	-0.9583045019	1.5903791661
H	-1.5453016759	-1.0989501372	-1.2695404989

7
TS of R28 in the main text

C	-0.4749503912	0.9478512778	0.0731354663
O	-0.1334288387	1.9787418023	-0.2675161564
H	-0.0969323345	-1.1055767236	0.3120880891
H	0.3814428792	-0.3703653820	0.4785487213

H	-2.7645004058	0.2418480380	-0.4832233257
C	-1.8811244232	-0.3202589238	-0.2349769291
N	-1.4552785158	-1.4220353486	-0.1135856055

9
TS of R29 in the main text

H	0.5354563151	1.1250024643	2.1427282731
C	0.7763908892	2.1659101417	2.0393407253
N	0.7730805626	3.1587346207	2.6797698311
O	1.7310127414	3.4537185230	-0.1402405997
C	1.3990722267	2.2780954795	0.1283180936
H	0.4347203075	1.8964912634	-0.2330782086
H	2.1790807427	1.5280214816	0.3158315931
H	1.2765543117	4.2931129657	1.8006009574
H	1.5338032532	4.3045711802	0.9946954148

8
TS of R30 in the main text

C	-1.6610569989	0.8687807471	0.1944982419
O	-1.9935374239	1.9168634700	0.4955259940
O	-0.8950409897	0.7337030171	-1.5136806771
H	-1.5234108786	1.0426065221	-2.1761031885
H	-0.9962756894	-0.3276786816	-1.3893749481
H	-1.9170059054	-1.0001807543	1.4868211220
C	-1.6187923160	-0.8557225972	0.4524940005
N	-1.3045948481	-1.5096162531	-0.5096067646

12
TS of R31 in the main text

C	-1.0870225432	-0.7529692306	-2.6755856116
N	-1.0400594297	-1.9338498761	-2.4463049457
H	-1.1023028916	-0.1557588379	-3.5808556274
H	-1.1510615003	-0.0153331732	-1.6440850027
H	-1.1812519724	-2.0007323740	-1.0828053639
C	-0.2890968565	0.8232385825	0.2539635572
C	-1.3063521505	-0.1885919641	-0.1495239854
N	-1.0596391019	-1.4952233105	-0.0136684423
O	-0.5349444348	1.9836490026	0.4415152867
H	-0.0763338552	-1.6605619630	0.2027171334
H	0.7403170625	0.4217712362	0.3714519415
H	-2.3405231564	0.1495010583	-0.1221144099

12
TS of R32 in the main text

C	-0.9454340980	1.5776618853	2.1198637135

N	-0.9562003370	2.6777144552	1.6507942389
H	-0.6707558678	0.6384661910	1.2112058650
H	-1.0948581740	1.0906280407	3.0734308964
H	-0.6897216758	2.4920934965	0.4425197129
C	-0.3867368066	0.5703459395	-0.1389393335
O	-0.4281558442	1.7990817598	-0.5198506207
H	-0.5461004042	-1.9517215388	-0.2183013758
H	0.5992153048	0.0893430834	-0.0392873647
H	-2.3868412588	0.1522720318	-0.9408051138
C	-1.4884337622	-0.3521498512	-0.5795080731
N	-1.4444640363	-1.6140188632	-0.5754480351

14

TS of R33 in the main text

O	-1.1456180466	2.9407416503	-0.5756800434
C	-1.4601228149	2.5824223392	0.5765544910
O	-1.8482187662	3.0469963347	1.5989491541
H	-0.7656627068	1.9314671647	-1.1317970215
H	-1.2994509504	1.3181627063	0.6348556589
C	-0.7632072538	0.2321697648	-0.1451392325
C	-1.8805777400	-0.7872437566	-0.0356159785
N	-1.3939096554	-2.1208670849	-0.3511768903
O	-0.5171157325	0.7834838282	-1.2801919604
H	0.0854237332	0.0621368798	0.5357910507
H	-0.7228746311	-2.4492435059	0.3311878778
H	-2.6672390303	-0.5090512244	-0.7381002126
H	-2.3012639099	-0.7888120939	0.9710531080
H	-0.9677615151	-2.1532402820	-1.2685594915

14

TS of R34 in the main text

O	3.3417944688	2.1149818004	3.7128860849
C	3.6488168116	1.3604704065	2.7726858772
O	4.4972068392	0.5320897031	2.5433277311
H	1.9640176094	2.9278928361	3.3379941239
H	2.8715354744	1.4947573127	1.8433146190
C	1.2650277162	2.1277738204	1.6940809697
C	1.0721124036	2.3108782327	0.2161462703
N	1.4015959715	3.1647561884	2.4764292802
O	1.3992660203	3.6239575214	-0.1815474872
H	0.9593898530	1.1873674683	2.1378700526
H	0.0381322759	2.0491991862	-0.0274786841
H	1.7163252141	1.6057760232	-0.3107360440
H	1.6714835499	4.0143035546	1.9913831055
H	0.5922957923	4.1207959460	-0.3443558993

17

TS of R35 in the main text

N	-0.3374009836	-0.4867539470	2.6934517410
H	0.4985128155	-0.0988300544	3.1255473508
H	-0.0843625649	-0.8054226368	1.7023919017
H	-0.6438104706	-1.3093960232	3.2091831557
O	-2.6008620698	-0.0732009255	2.1469314807
C	-1.5145715985	0.5209630268	2.6605658234
H	-1.1131681601	1.3569047997	2.0777371615
H	-1.6625221590	0.8184576536	3.7017854356
C	0.4948963156	-0.9325620623	-0.0860965531
C	-0.8565287159	-1.0744762223	-0.2529669236
N	-1.8069834728	-0.1284420875	-0.2481606604
O	1.0586859899	0.3532363265	0.0621524676
H	-1.3591208082	0.7853096991	-0.3083527319
H	1.1171307550	0.7749680132	-0.8026831823
H	1.1962806395	-1.7433392887	-0.2357973061
H	-1.2076192032	-2.1079883923	-0.2967779547
H	-2.4319304989	-0.1358982990	1.0606806241

14

TS of R36 in the main text

O	1.1530322635	-0.6921527846	2.0513353420
C	-0.0711172184	-0.7714148739	2.2803625314
O	-0.7638316161	-0.7250684010	3.2790657847
H	1.1676666502	-0.9722122015	0.3892651337
H	-0.6921868709	-0.9248280452	1.2890552694
C	0.8259768612	-0.8469506827	-0.6807557314
C	-0.6340533895	-0.9686219721	-0.6489519368
N	-1.3733513512	0.0236683740	-1.0586776798
O	1.1972436856	0.4217617807	-1.1853833685
H	-0.8953038156	0.8877425940	-1.2879538880
H	1.7254737785	0.8591653340	-0.5078070744
H	1.2458476353	-1.6618865858	-1.2795902158
H	-1.1014644905	-1.9277745789	-0.4711920494
H	-2.3779321221	-0.0074279571	-1.0347721171

16

TS of R37 in the main text

O	0.1671814054	-2.8105912730	-0.9423678129
H	-0.6643187047	-3.0790951706	-1.3508938412
H	0.1069414810	-3.1010883817	0.0514050124
O	1.5702162601	-1.9727465101	1.9944748871
C	0.8993727215	-3.0204448803	2.2196612272
O	0.0567410049	-3.5446503165	1.4691734105
H	1.1952345052	-1.2462343444	0.8966571701
H	1.0935623884	-3.5069849645	3.1897525409
C	1.1572339758	-0.3533093885	-0.0129986291
C	0.3134828231	-0.8381602834	-1.0143816360
N	2.5363865957	-0.0364046366	-0.3424343681

H	0.6725669672	0.4210805927	0.5859907458
H	0.6726187965	-0.9650038423	-2.0271104047
H	-0.7507675520	-0.6926923167	-0.9064781384
H	2.8683492983	-0.6295365714	-1.0925193602
H	3.1249395737	-0.2344061926	0.4578145767

17

TS of R38 in the main text

N	-0.6467295588	-2.0548750171	-1.0816102255
H	-1.3968007864	-1.9731419172	-0.4050571385
H	0.0874507372	-2.6730322894	-0.6973494312
H	-1.0170402531	-2.4311065438	-1.9472820549
O	1.0196487584	-1.7648158601	1.7032528977
C	1.3509139495	-2.9700844019	1.3923361387
O	1.3374260378	-3.4683652273	0.2705006705
H	0.9211844426	-1.0131025948	0.7794275854
H	1.6653126715	-3.5739273047	2.2585571197
C	1.1173821464	-0.0355916198	-0.2512517171
C	0.2150108808	-0.3362422335	-1.2818352064
N	2.5455753433	-0.1515125532	-0.4996130646
H	0.8417678539	0.8560258622	0.3122436740
H	0.5987939238	-0.6040641454	-2.2624174984
H	-0.7145653141	0.2160439063	-1.3189200396
H	2.7392009612	-0.4977252750	-1.4300426649
H	2.9874682061	-0.7864827855	0.1530609550

16

TS of R39 in the main text

N	0.7485606854	-2.7121166477	1.4996045619
H	0.6474751188	-2.1035739265	0.6084408867
H	0.4401896445	-3.6590568772	1.2849775908
H	0.1326870032	-2.3145232246	2.2080035884
O	2.4639454741	-1.4495377073	2.5470190659
C	2.1943863295	-2.6717980227	2.0738029461
H	2.2016393055	-3.4291583914	2.8627053158
H	2.8168541282	-3.0033609788	1.2327373001
C	1.2860331027	-0.1970646178	-0.0426844771
C	0.6312142829	-1.0086006996	-0.9429695325
N	2.5600589363	-0.2120589244	0.3565899549
H	0.6537033190	0.4905283388	0.5283096033
H	1.2005801268	-1.6530366628	-1.6090485876
H	-0.3879886325	-0.7813674360	-1.2288731070
H	3.1161166861	-0.7965094935	-0.2675101720
H	2.5927473895	-0.7793107183	1.6650978322

15

TS of R40 in the main text

O	-0.1560008464	2.0017236028	1.3967217408
C	0.2938806932	1.5032442363	0.2071737291
O	0.4425232995	2.2307450919	-0.7433603090
H	-0.2303644144	2.9658700661	1.2817811524
H	0.9848109812	0.2920348480	0.4424080320
O	1.6022702670	-2.1124883820	-0.6305632995
C	2.2625416188	-1.4581729870	0.1796576241
O	1.8905426289	-0.4153648353	0.8255549581
H	-0.0091858404	-1.9203585077	-0.2946513714
H	3.2859761079	-1.7790962432	0.4237747180
C	-1.0349164610	-0.2117939133	-0.4429984835
N	-0.8541529988	-1.4012294435	0.0391576625
H	-1.8285609050	0.4080956440	-0.0466366652
H	-0.6096617227	0.0244595921	-1.4075446403
H	-1.2731014378	-1.6526055891	0.9220631317

13
TS of R41 in the main text

C	3.6827680912	1.2042388223	0.3149920605
N	4.0331625489	0.5077194619	-0.6033796066
H	2.4628815318	1.0916630681	0.6270645396
H	4.1928457721	1.9134663057	0.9576684981
H	2.9158170734	-0.2332442828	-0.8930762776
O	-0.2595017317	1.7006963021	1.2453214576
C	0.3027227611	1.3721935862	0.0693705015
O	0.0655147511	1.9353479212	-0.9663112005
H	-0.8545611357	2.4453671362	1.0697269425
C	1.2689054098	0.2430013992	0.2087284078
N	1.7325693724	-0.3414414437	-0.8923178064
H	1.2307429468	-0.3194164144	1.1371639708
H	1.4501204487	0.2085050779	-1.7055389873

19
TS of R42 in the main text

O	3.1157576905	-1.6390736680	2.4817651954
C	2.0000826943	-1.5603305617	1.8737175496
H	1.2828196282	-0.8346666131	2.2458764052
H	1.6189724346	-2.4390027222	1.3481425320
O	4.1737455783	-3.4779748959	1.3148444571
C	4.0559134871	-3.2622735116	0.0895632481
O	3.4775445309	-2.2823898541	-0.4590207833
H	3.6701450405	-2.4883155534	2.0382814122
H	4.4939699100	-3.9980775890	-0.5958780936
C	0.6591836761	-0.2735478056	0.0019278862
O	-0.1941108791	0.1949169813	0.7200132529
N	2.5034517966	1.0022318696	0.9921841630
C	2.1212448016	-0.1365809020	0.1977270904
O	0.3701099672	-1.0772265811	-1.0423631851
H	1.7252050385	1.3027468009	1.5689366777

H	3.2876113523	0.7890672189	1.5949890493
H	2.8081828336	-1.3415763544	0.1347608817
H	-0.5939725239	-1.1684233872	-1.0676110581
H	2.5751429424	-0.0795028713	-0.8018566807

14
TS of R43 in the main text

H	3.2810535335	-0.4949939776	-0.9952973558
H	3.8916143461	0.1481350466	-1.0458852255
O	4.3382245482	1.2757299443	-0.3389446051
C	3.4346276158	1.8336030794	0.3129365528
H	3.4204467367	1.7652727411	1.4041925169
H	2.8349516485	2.6186058404	-0.1576911954
O	0.3937240923	2.0854616876	1.1563980915
C	0.7479580096	1.4105693361	0.0442794116
O	0.3338214278	1.6770727401	-1.0552133606
H	-0.2355243749	2.7682312413	0.8799464689
C	1.6876332469	0.3069239827	0.3464625217
N	1.9720019632	-0.5694592954	-0.5878844897
H	1.8578256215	0.0480677792	1.3842349673
H	1.5655249648	-0.2501831859	-1.4741618785

19
TS of R44 in the main text

O	3.6495111415	0.1982737398	2.6463822142
C	3.6954850759	1.5822303649	2.5962365761
O	4.4337543593	2.0992040871	1.7865614911
H	4.3301015102	-0.1243798764	2.0272226806
H	3.3290655950	2.2891809571	3.9657791706
O	1.9847791787	4.3743954139	4.6839129562
C	2.8013672874	3.7342752711	5.3391716948
O	3.4352533340	2.6677980791	4.9922814131
H	1.6191765589	4.0727983064	3.0921991787
H	3.0580982529	4.0584534826	6.3564482170
C	1.4665588569	2.3282821845	2.1174399545
C	1.3996777559	1.6498252732	0.7739866310
N	1.5173984502	3.6162846107	2.1619585943
O	1.4756630804	2.5842584259	-0.2810764967
H	1.2040042542	1.7786274634	3.0106227785
H	0.4752291882	1.0651497428	0.7421612504
H	2.2298369255	0.9568841474	0.6828478710
H	1.7724670888	4.0912608805	1.3049263025
H	0.5854937362	2.8623143661	-0.5184168579

14
TS of R45 in the main text

C	-1.2869847364	-0.6783677746	2.0407449785

O	-1.5086927758	0.4335357224	2.2775156774
O	0.4785956994	-1.0073722540	2.5609366765
H	0.6575301058	-1.0570515837	0.9890528646
H	0.4522750900	-1.9298545673	2.8418772608
C	0.5646794712	-0.8309498500	-0.1543297338
C	-0.8273239035	-1.0903900576	-0.4644695593
N	-1.5906558622	-0.1578928682	-0.9484569571
O	0.8999204185	0.5130525309	-0.4371254535
H	-1.1906971297	0.7717246342	-1.0142083831
H	1.1569829057	0.9212254691	0.3990077085
H	1.2118449544	-1.5416785715	-0.6811474242
H	-1.2305039264	-2.0864321187	-0.3502303801
H	-2.5815008710	-0.2865527710	-1.0625903352

18

TS of R46 in the main text

O	-3.2927372454	-2.9206021157	2.1611235657
C	-3.3891422529	-2.2469732316	0.9695851430
O	-4.2728318222	-1.4267145698	0.8305684133
H	-4.0480159749	-2.6335575031	2.7059073307
H	-2.7474942014	-3.0180342420	-0.1715271017
O	-2.0446982942	-2.3462650674	-2.5949840004
C	-2.3019433190	-3.4687073233	-2.1714020662
O	-2.5496561519	-3.8180597688	-0.9572360231
H	-1.8948168230	-1.0164343777	-1.5931708431
H	-2.3251374400	-4.3178361705	-2.8696632343
C	-1.4378569394	-1.0185205570	0.3514817039
C	-1.4191419047	-0.3167225814	1.6644396111
N	-1.8465452377	-0.4367607987	-0.7293042342
H	-0.8631245846	-1.9278107869	0.2262344337
H	-1.4813396592	-1.0311163852	2.4791251394
H	-2.2467959156	0.3876017245	1.7352435794
H	-0.4753719737	0.2300871002	1.7431527821
H	-2.4265019804	0.3867833245	-0.6525744692

13

TS of R47 in the main text

C	0.5630104946	-2.0682439474	1.4974994398
O	0.5991201485	-3.0927215931	2.0421264065
O	-0.9408133867	-1.9404592908	0.4685630236
H	-1.2360620965	-2.8514280189	0.3477246889
H	-0.1152295815	-1.1908438538	-0.5101248500
C	1.4587021383	-0.0509497891	0.1402319681
C	0.6263712217	-0.3627470797	-0.9780166059
N	2.7557348032	-0.2549531022	0.1918814188
H	1.0326384448	0.4726168816	0.9844253488
H	1.1599283923	-0.7400320669	-1.8479197198
H	-0.0616552320	0.4477072348	-1.2142929587
H	3.2161351814	-0.7787793824	-0.5352130397

H	3.2464865919	-0.1499043120	1.0634303596
